# Multistep magnetization switching in orthogonally twisted ferromagnetic monolayers

**DOI:** 10.1038/s41563-023-01735-6

**Published:** 2023-11-30

**Authors:** Carla Boix-Constant, Sarah Jenkins, Ricardo Rama-Eiroa, Elton J. G. Santos, Samuel Mañas-Valero, Eugenio Coronado

**Affiliations:** 1 Instituto de Ciencia Molecular (ICMol) - Universitat de València, Paterna, Spain; 2 Institute for Condensed Matter Physics and Complex Systems, School of Physics and Astronomy, The University of Edinburgh, Edinburgh, UK; 3 Donostia International Physics Center (DIPC), Donostia-San Sebastián, Spain; 4 Higgs Centre for Theoretical Physics, The University of Edinburgh, Edinburgh, UK; 5 Kavli Institute of Nanoscience, Delft University of Technology, Delft, The Netherlands

**Keywords:** Two-dimensional materials, Magnetic properties and materials

## Abstract

The advent of twist engineering in two-dimensional crystals enables the design of van der Waals heterostructures with emergent properties. In the case of magnets, this approach can afford artificial antiferromagnets with tailored spin arrangements. Here we fabricate an orthogonally twisted bilayer by twisting two CrSBr ferromagnetic monolayers with an easy-axis in-plane spin anisotropy by 90°. The magnetotransport properties reveal multistep magnetization switching with a magnetic hysteresis opening, which is absent in the pristine case. By tuning the magnetic field, we modulate the remanent state and coercivity and select between hysteretic and non-hysteretic magnetoresistance scenarios. This complexity pinpoints spin anisotropy as a key aspect in twisted magnetic superlattices. Our results highlight control over the magnetic properties in van der Waals heterostructures, leading to a variety of field-induced phenomena and opening a fruitful playground for creating desired magnetic symmetries and manipulating non-collinear magnetic configurations.

## Abstract (web summary)

The authors present magnetotransport measurements to demonstrate multistep magnetization switching in orthogonally twisted CrSBr ferromagnetic monolayers.

## Main

Metamagnets and their field-induced phase transitions offer a plethora of counterintuitive phenomenology, as quoted by Kramers^1^, with direct competition between magnetic anisotropy, exchange and dipolar energies^2^. In absence of a magnetic field, these materials show zero net magnetization that suddenly increases until its saturation—thus resembling a ferromagnet—above a certain magnetic field threshold^1^. A good example of an A-type metamagnet is offered by the layered van der Waals (vdW) semiconductor CrSBr. The spins in every single layer (*a*–*b* plane) couple ferromagnetically between them (*T*_C_ ≈ 150 K, *T*_C_ is the Curie temperature), pointing along the easy *b* axis, whereas the layers couple between them antiferromagnetically (*T*_N_ ≈ 140 K, *T*_N_ is the Néel temperature)^3^. By applying a magnetic field, it is possible to flip the layers’ magnetization in a parallel fashion via a spin reversal and induce a spin reorientation along the magnetic field direction. This transition does not present hysteresis^3–11^. In bulk, the saturation fields at 2 K are 0.6 T, 1 T and 2 T for the easy (*b*), intermediate (*a*) and hard (*c*) magnetic axes, respectively^9^. This vdW material can be thinned down to the monolayer limit and integrated into electronic nanodevices. Upon field-induced spin switching, the magnetoresistance (MR) is large and negative from the bulk down to the bilayer case, with a reduction of the saturation field along the easy axis (from 0.6 T in bulk to 0.2 T in the bilayer at 2 K)^7–9,12,13^. The monolayer limit is characterized by the absence of MR for fields applied along the easy axis and small and positive MR for fields applied along the intermediate and hard axes^9,12^.

The ability to isolate, manipulate and twist two-dimensional (2D) crystals adds a new degree of control in vdW heterostructures, affording emergent new properties, such as superconductivity in twisted bilayer graphene^14^. As far as the 2D magnetic materials are concerned, twisting is much less explored. Still, it has allowed the creation of new magnetic ground states. For example, by twisting the 2D magnet CrI_3_ by small angles, modulation of spin reversal by magneto-optical techniques has been reported^15–17^. This twist engineering not only produces a moiré superlattice but also can induce moiré magnetic exchange interactions, in which unique spin textures such as a magnetic skyrmion have been theoretically predicted^18–21^. However, no 2D twisted magnets have been incorporated into electronic devices so far and the magnetotransport effects in twisted magnets remains fully unexplored.

**Fig. 1:**
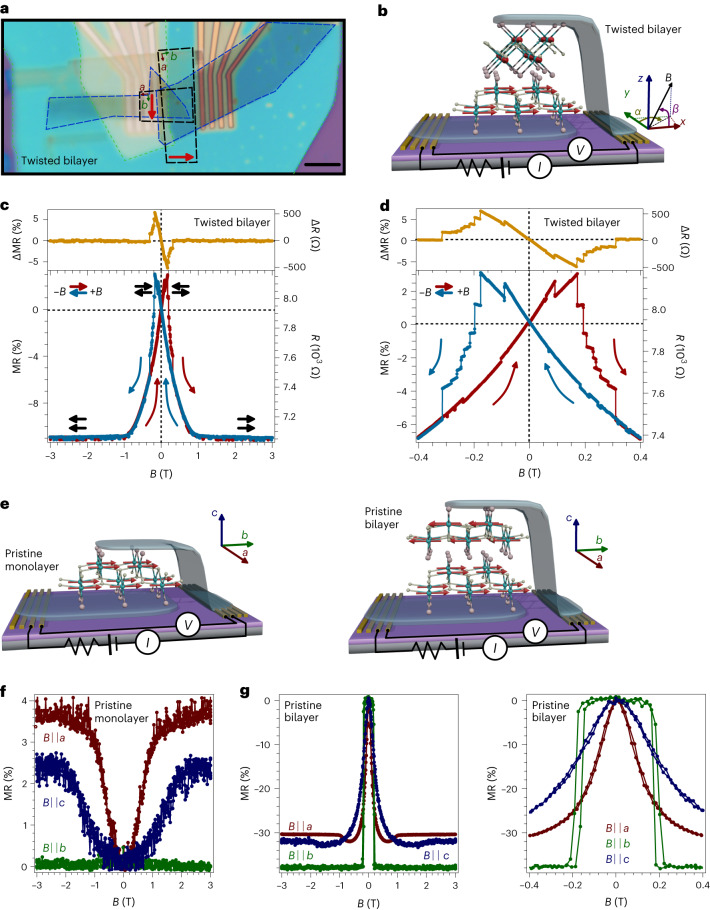
Magnetic field dependence of the MR in orthogonally twisted bilayer CrSBr. **a**, Optical image of a vertical vdW heterostructure consisting of twisted CrSBr monolayers (black dashed lines) in between few-layer graphene (blue dashed lines). Different insulating hexagonal boron nitride layers (green dashed lines) are used both to avoid shortcuts and to protect the heterostructure. The red arrows indicate the easy magnetic axis (*b*) of each CrSBr monolayer, with the intermediate magnetic axis (*a*) perpendicular to it. The hard magnetic axis (*c*) corresponds to the out-of-plane direction. Scale bar, 5 µm. **b**, Schematic view of the heterostructure (not to scale), highlighting the twisted CrSBr monolayers placed in between few-layer graphene or NbSe_2_ thin layers (blue) on top of pre-patterned electrodes (gold) together with a sketch of the electrical measurement configuration (I refers to current and V to voltage). Pink, yellow and cyan balls correspond to bromine, sulfur and chromium atoms, respectively. The red arrows represent the spin lying along the easy magnetic axis, assuming negligible interlayer magnetic interactions. **c**,**d**, Field dependence of the resistance (*R*) and the MR (bottom) and its increment (top), defined as Δ*X* = *X*_+*B*→−*B*_ − *X*_−*B*→+*B*_, where *X* indicates either the resistance or the MR at *T* = 10 K and *θ* = *φ* = 0º. The sweeping up (down) trace is depicted in red (blue). The red and blue arrows indicate the sweeping direction of the magnetic field. The black arrows sketch the relative configuration of both layers’ magnetization. MR is defined as MR (%) = 100[*R*(*B*) – *R*(0)]/*R*(0), where *R*(0) is the resistance obtained at zero field. Panel **d** shows a zoom of panel **c**. **e**–**g**, Reference experiments on pristine monolayer CrSBr and pristine bilayer CrSBr based on our previous work^9^, including the corresponding sketches (**e**) and field dependence of the MR for fields applied along the easy (*b*), intermediate (*a*) and hard (*c*) magnetic axes for pristine monolayer (**f**) and bilayer (**g**, left (right) figure shows a broad (small) field range) at *T* = 10 K.

Here we twist two CrSBr ferromagnetic monolayers by about 90°, thus forming an orthogonally twisted bilayer. In analogy to the artificial antiferromagnets reported in synthetic spintronics—where the magnetic properties are tailored by growing multilayers of different antiferromagnets, in contrast to crystalline bulk antiferromagnets^22^—this twisted heterostructure can be envisaged as an artificial antiferromagnetic bilayer. To probe its magnetotransport properties, this bilayer is integrated in a vertical vdW heterostructure formed by either few-layer graphene or metallic NbSe_2_ thin layers (Fig. 1a,b and Methods)^23–26^. Note that, in stark contrast to CrI_3_, where the spins are out-of-plane, in CrSBr the spins are in-plane, pointing along the easy magnetic *b* axis, with an intermediate *a* axis (also in-plane) but with a hard magnetic *c* axis (out-of-plane direction). This orthogonal configuration yields an intriguing spin scenario where several terms might compete with an applied magnetic field such as the Zeeman split energy, the interlayer magnetic interactions (which favours an antiparallel orientation between the layers) and the local spin anisotropy in each CrSBr layer (which are perpendicular in the twisted configuration). This case is different from the common moiré patterns in twisted bilayers, where a modification of the band structure is reached by twisting by small angles^14^.

An example of an orthogonally twisted CrSBr heterostructure is shown in Fig. 1a,b. In this vertical geometry, the MR can be rationalized within a spin-valve picture, considering a two-current channel model: when the magnetization of both layers is antiparallel (parallel), there is a higher (lower) resistance across the heterostructure^9,27,28^. The field dependence of the MR at 10 K is presented in Fig. 1c for in-plane magnetic fields aligned along the easy axis of one of the layers (in this case, the top layer; *α* = *β* = 0°, as defined in Fig. 1b). Starting at high negative fields (red curve in Fig. 1c), the MR is negative and field independent down to −1 T; then it increases until a maximum positive MR is observed at about +0.16 T. Above this field, it decreases again until reaching a saturation value above +1 T. This value coincides with that observed for the spin reorientation along the intermediate magnetic axis, *a*, thus suggesting that this is determined by the spin anisotropy. Reversing the magnetic field yields a symmetrical curve that exhibits maximum MR at about −0.16 T (blue curve in Fig. 1c). These two curves cross at zero field, showing a hysteretic behaviour when the field modulus is kept below about 0.32 T. For an easier visualization of the hysteresis, in Fig. 1c (top) we present the increment value, defined as Δ*X* = *X*_+*B*→−*B*_ − X_−*B*→+*B*_, where *X* indicates either the resistance (*R*) or the MR while decreasing (+*B* → −*B*; blue curve in Fig. 1c) or increasing (−*B* → + *B*; red curve in Fig. 1c) the external magnetic field (*B*). Then, non-zero Δ*X* values indicate a hysteretic effect. In addition, a zoom of the hysteretic region is presented in Fig. 1d, showing several resistance drops and plateaus and two lower limiting MR branches (with positive (red) and negative (blue) slopes) crossing at zero field. No relevant influence of the field sweeping rate is observed (Supplementary Fig. 1). For a better comparison with the orthogonally twisted bilayer, we show the corresponding MR behaviour for pristine monolayer and pristine bilayer CrSBr in Fig. 1e–g^9^. In the pristine case, the spin reversal takes place via a spin flip for fields applied along the easy magnetic axis and a spin-canting process for fields along the intermediate and hard magnetic axes^6,7,9,12^.

A qualitative understanding of the MR behaviour of the orthogonally twisted bilayer upon the application of a magnetic field along the easy (intermediate) magnetic axis of the top (bottom) monolayer (Fig. 1c) is as follows: at high negative fields (region from −3 T to −1 T), the magnetization of both layers is parallel (*φ* = 0°, where *φ* is the angle formed between the magnetization of the top and bottom layer) and yields a state of low resistance according to the spin-valve picture. Below −1 T, the anisotropy is able to progressively reorient the magnetization of the bottom layer from its intermediate magnetic axis towards its easy magnetic axis, while that of the top layer stays unchanged as the field is applied along its easy magnetic axis. As a consequence, from −1 T to 0 T, an increase of the resistance is observed in agreement with the progressive increase of *φ*. In fact, at zero field, the magnetization of both layers would be orthogonal (*φ* = 90°), assuming negligible interlayer interactions. Upon the application of positive fields, the magnetization of the bottom layer continues the canting process (*φ* > 90°), tending to adopt an antiparallel configuration to satisfy the antiferromagnetic coupling, thus increasing the resistance to a maximum value at 0.16 T. At this point, the top layer flips its magnetization to be oriented along the positive magnetic field and *φ* decreases (*φ* < 90°), thus yielding a big drop in the resistance. Further magnetic fields tend to continue canting the magnetization of the bottom layer, thus decreasing *φ* and, therefore, the resistance. Above 1 T (range from 1 T to 3 T), the magnetization of the top and bottom layers is parallel (*φ* = 0°) and the lower resistance state is observed. Decreasing magnetic fields give rise to a symmetric configuration but the MR peak appears at negative field values and, consequently, yield a hysteretic effect (a detailed view of the process is presented in the Supplementary Fig. 2). This scenario, which is possible owing to the in-plane magnetic anisotropy of CrSBr, cannot be observed in twisted CrI_3_, as it shows out-of-plane anisotropy. This behaviour is in sharp contrast to that of pristine bilayer CrSBr, which shows a single maximum of MR at zero field, as a result of the antiparallel orientation between the two layers, and no hysteretic effects (Fig. 1e–g)^9,12^. Finally, we consider the in-plane angular dependence (Supplementary Fig. 3). All the curves show the same general trend discussed above but with different coercivity fields and Δ*X* values. The field orientation hence allows for a fine tuning and control of the hysteretic parameters. Note the asymmetry between 0° and 90°, which indicates that the underlying spin dynamics are dominated by one of the layers—as discussed later, this is due to the larger stray fields at the twisted layers. We note that for fields applied along directions different to that of the easy magnetic axis, the reversal mechanism can be more complex as both layers can be canting, thus motivating future magnetic imaging experiments in these CrSBr twisted layers. Regarding magnetic fields applied along the hard magnetic axis *c* (out-of-plane direction), a hysteretic behaviour is manifested as well, but with a notable broader maximum of MR (Supplementary Fig. 4). In this case, the MR curves are saturating for fields above 2 T, which, as for the in-plane case, coincides with the field needed to reorient the spins along the magnetic field direction (*c* in the present case; Fig. 1e–g). Similar results are observed in different orthogonally twisted bilayer CrSBr heterostructures, underlying the robustness of the observed phenomenology (Supplementary Fig. 5), although the exact switching magnetic values differ between the different devices, probably due to slightly different twisting angles.

**Fig. 2:**
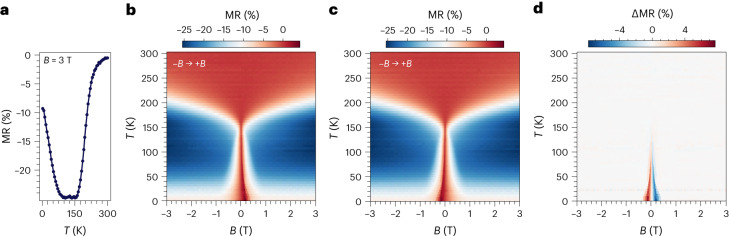
Field and temperature dependence of the MR in orthogonally twisted bilayer CrSBr. **a**, Temperature dependence of the MR at saturated fields (*B* = 3 T). **b**,**c**, Field and temperature dependence of the MR while sweeping from negative to positive (**b**) and from positive to negative (**c**) fields. **d**, Field and temperature dependence of ΔMR. MR is defined as MR (%) = 100[*R*(*B*) – *R*(0)]/*R*(0) and ΔMR = MR_+*B*→−*B*_ − MR_−*B*→+*B*_*; θ* = *φ* = 0°.

Next, we consider both the field and temperature dependence of the MR (Fig. 2). We observe that the behaviour resembles that reported for the pristine bilayer^9^. Upon cooling the system, a negative MR starts developing below 200 K due to the onset of short-range interactions within the layers. Then MR reaches a broad plateau at about 150 K, near *T*_C_, and below 100 K it increases again (Fig. 2a). However, some differences to the pristine bilayer are observed. First, in the pristine bilayer, a minimum in MR, instead of a plateau, is observed at 150 K, followed at 100 K by a decrease. Second, a hysteretic behaviour is observed for temperatures below *T*_N_ (Fig. 2b–d), increasing the coercive field and ΔMR upon cooling, while no hysteresis is observed in the pristine bilayer. Similar trends are observed for fields applied along different directions (Supplementary Fig. 6).

**Fig. 3:**
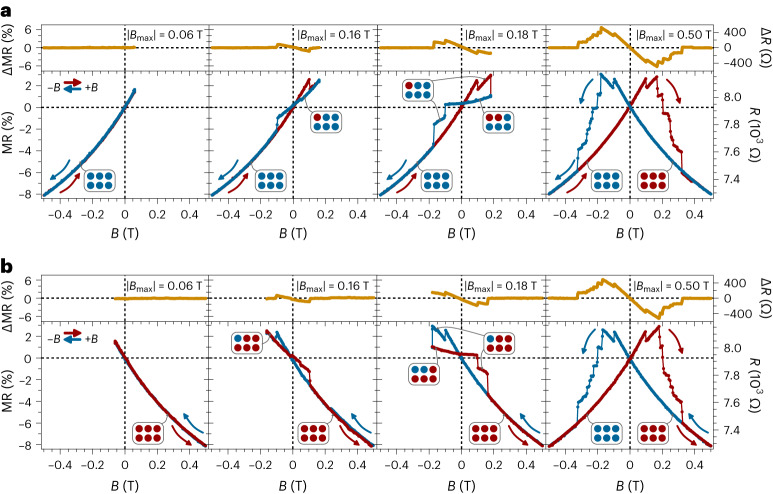
Multistep magnetization switching with magnetic memory in orthogonally twisted bilayer CrSBr. **a**,**b**, FORCs considering the sequence −0.6 T → +*B*_max_ → −0.6 T (**a**) and +0.6 T → −*B*_max_ → +0.6 T (**b**) at 10 K and *θ* = *φ* = 0°. *B*_max_ is incremented sequentially in steps of 20 mT and selected curves are shown (see Supplementary Video 1 for the whole dataset). The saturated state at negative (positive) magnetic fields is schematically sketched as a set of blue (red) circles, with each spin switch related to the change of one individual hysteron (squared hysteresis operator characterized by a coercive field and a field shift from zero) within the Preisach model. MR is defined as MR (%) = 100[*R*(*B*) − *R*(0)]/*R*(0).

To further explore the irreversibility of the observed hysteresis in Fig. 1, we perform a series of first-order reversal curves (FORCs). FORC analysis lies behind the Preisach model^29,30^. We increment sequentially the maximum applied magnetic field (*B*_max_) in steps of 20 mT, after an initial saturation at negative fields (sequence: −0.6 T → +*B*_max_ → −0.6 T). Selected curves are shown in Fig. 3a (see Supplementary Video 1 for the whole dataset). For sweeping fields below about 0.1 T (|*B*_max_| = 0.06 T in Fig. 3a), the resistance increases (decreases) with increasing (decreasing) *B* following the behaviour already observed in Fig. 1d when sweeping from negative fields (limiting branch with positive slope). No hysteresis is observed for this loop, with the MR curve being symmetric (ΔMR = 0 at zero field). A more interesting scenario is offered when this field threshold is overcome (|*B*_max_| = 0.16 T in Fig. 3a). In this case, the resistance increases (red curve) on increasing *B*, as before, until a sharp drop occurs at about 0.1 T. Then, on decreasing *B* (blue curve), the resistance decreases but with a smaller slope until a second drop is observed at about −0.1 T, when it returns to the initial path (limiting branch with positive slope). This behaviour results in the emergence of an asymmetric hysteresis (ΔMR ≠ 0 at zero field). Similar asymmetric curves with successive drops in the resistance, giving rise to steps and plateaus at well-defined magnetic fields, are observed on increasing the maximum sweeping magnetic field value (|*B*_max_| = 0.18 T in Fig. 3, Supplementary Video 1 and Supplementary Fig. 7). Interestingly, each step observed for positive fields is characterized by a different slope while returning to zero field. This slope decreases until a saturation field is reached (0.32 T in the present case). For *B* > 0.32 T, the limiting branch with negative slope is reached and the hysteresis loop becomes fully symmetric with respect to the *R* axis (|*B*_max_| = 0.50 T in Fig. 3a). Interestingly, when coming from positive saturated fields (sequence: +0.6 T → −*B*_max_ → +0.6 T in Fig. 3b and Supplementary Video 1), the same phenomenology is observed but reversing the modulus of the switching fields (mirror image with respect to the *R* axis).

Therefore, this magnetic system is formed by two ground states that are degenerated at zero field but that evolve with opposite MR slopes in the presence of *B*. Thus, an initial saturation at negative fields leads to a state defined by the MR branch with positive slope (Fig. 3a). This state is sketched as a set of blue circles in the Fig. 3. Conversely, when coming from positive fields, a different state is obtained (set of red circles in Fig. 3) leading to the MR branch with negative slope. For *B* values within the range ±0.32 T, the system evolves hysteretically and selectively towards one of these two ground states and only for higher |*B*| values is a change of ground state possible. This allows to select at will the ground state of the system. Furthermore, in the hysteretic region, such evolution takes place through successive steps at specific fields that may be associated with intermediate states. This multistep phenomenology can be related to the Preisach model. Thus, starting from one of the two MR branches, each of the resistance drops observed in the hysteresis curves is associated with the switch of an individual hysteron, leading to each of the intermediate states postulated above. In applied terms, each of these switches could be potentially employed as a bit of information. This is schematically sketched in Fig. 3 by sweeping red and blue bytes. Importantly, there is also magnetic memory at zero field as we can select between hysteretic and non-hysteretic MR scenarios depending on the initial ground state of the system. In the Supplementary Fig. 8, we consider different magnetic field sweep protocols and, for example, in the sequence zero field → +*B*_max_ → zero field we observe hysteresis only after an initial saturation in negative magnetic fields. Therefore, the magnetic history allows us to control the appearance or not of hysteresis. To manifest the robustness of these results, we present in Supplementary Fig. 9 the study for other orthogonally twisted CrSBr bilayers. Overall, similar trends, although at different switching fields, are observed under different in-plane field orientations (Supplementary Fig. 10) and temperatures (Supplementary Fig. 11).

**Fig. 4:**
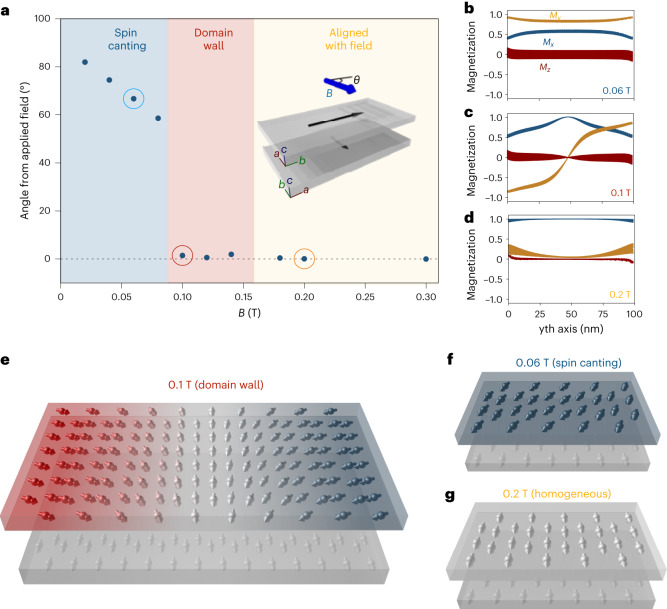
Field-induced spin textures in orthogonally twisted bilayer CrSBr. **a**, After cooling under different applied field strengths (0.01–0.3 T) applied along *x*—parallel (perpendicular) to the easy (intermediate) magnetic axis of the bottom (top) layer—we calculate the angle *θ* between the magnetization direction (**M/M**_s_) of the top layer to the applied field **B** (blue arrow). The field is initially applied along one of the easy axes of the layers (dark arrows), and as its magnitude increases **M/M**_s_ changes accordingly to be aligned with **B** (see inset). Three magnetic phases can be stabilized with the applied field: spin canting (the spins are aligned but at an angle between the anisotropy easy-axis direction of the top layer and the applied field direction), domain wall (part of the spins of the top layer orient along the field and the other part with the bottom layer underneath, which induces the formation of domain walls) and homogeneous (both layers have their spins aligned with the field). Three values of the field (0.06 T, 0.1 T and 0.2 T) are highlighted with circles and further analysed in the following panels as an example. The crystallographic *a*, *b* and *c* axes for each monolayer are indicated. **b**–**d**, Projections of the magnetization *M*_*x*_, *M*_*y*_ and *M*_*z*_ at 0 K as a function of the position (nm) along the *a* axis of the top layer at 0.06 T (**b**), 0.1 T (**c**) and 0.2 T (**d**). **e**–**g**, Schematics of the spin configuration observed in the spin dynamics simulations (Supplementary Fig. 12) at 0.1 T (domain wall; **e**), 0.06 T (spin canting; **f**) and 0.2 T (homogeneous; **g**).

The origin of the multistep magnetization switching can be attributed to the stabilization of different domain configurations and spin textures as revealed by atomistic spin dynamic simulations. We have considered the case of a CrSBr-based orthogonally twisted bilayer, where the top monolayer is rotated 90° with respect to the bottom one (inset in Fig. 4a). The size of the simulation system is 100 nm × 100 nm along the *x* and *y* axes, with no periodic boundary conditions along the aforementioned directions, and a cell thickness along the *z* axis corresponding to two unit cells to accommodate the two stacked monolayers (see Methods for details). In line with the experimentally based measurement protocol, we apply a simulated field of varying strengths along the *x* axis, that is, along the easy (intermediate) magnetic axis of the bottom (top) layer. Note that for an easier visualization of the results, the easy (intermediate) magnetic axis of the bottom (top) layer is rotated compared with Fig. 1a–d. We then simulate the field cooling from 200 K (above *T*_N_) to 0 K using spin dynamics techniques for 2D magnets^31–33^. In this way, it is possible to follow microscopically the variation of the magnetic features at the final simulated state of the system for different field strengths. We evaluate the angle *θ* between the magnetic moment vector **M**/*M*_s_ (where *M*_s_ is the volumetric saturation magnetization) of the top monolayer and the *x* direction, as it provides a strong descriptor of the spin orientations at the layers. Interestingly, we have observed that when low fields are applied (0.01–0.095 T), the magnetization of the top monolayer is canted from its easy *b* axis towards its intermediate *a* axis (Fig. 4a,b). If we increase the magnitude of the magnetic field to 0.10–0.14 T (Fig. 4a,c), we observe the appearance of non-collinear spin configurations in the form of hybrid domain walls (Bloch type) in the top monolayer. Intriguingly, this type of magnetic configuration, for this range of field strengths, occurs only if chiral spin interactions such as Dzyaloshinskii–Moriya interactions (DMIs) are considered in the simulations^34^, causing a magnetic frustration due to the competing contributions. This could lead to the appearance of more complex non-collinear spin distributions for larger systems. When the applied field is increased further to 0.18–0.30 T (Fig. 4a,d), the Zeeman-like contribution will overpower the internal fields causing the magnetization of the top monolayer to align along the magnetic field direction, that is, along its intermediate magnetic axis, *a*. An illustration of each spin phase at specific field magnitudes is shown in Fig. 4e–g, with the snapshots extracted from the simulations in Supplementary Fig. 12. Supplementary Videos 2–4 show the entire evolution of the dynamics at 0.06 T, 0.10 T and 0.20 T, respectively. For instance, we observed that at 0.10 T (Fig. 4e and Supplementary Video 3), the domain wall profile of the top layer flips from the +*y* to *−y* direction and the spins at the centre are along the applied field direction, *x*, parallel to the bottom monolayer. We observed that these different spin textures are not present on the pristine bilayer CrSBr as expected, as both layers have their easy axes along the same direction. Moreover, we have applied temperature to the system (5 K) and the simulated results remain consistent despite the thermal fluctuations and noise (Supplementary Fig. 13). It is worth mentioning that as one of the layers is twisted (for example, the top layer), the dipolar fields *H*_dip_ generated at that layer become larger relative to the untwisted layer (for example, the bottom layer), which conditionate the response of the system (Supplementary Fig. 14). That is, one of the layers becomes more dominant than the other, inducing the appearance of some of the MR effects discussed before in the measurements. Indeed, the variations of stray fields with the applied field follow those observed in the spin textures with the formation of canting fields and domain walls (Supplementary Fig. 12). This suggests that the dynamic evolution of the magnetization with the external magnetic field follows a Barkhausen-like effect trend^35^ with a series of sudden changes in the size and orientation of the magnetic domains. In our case, however, as the top layer is twisted with respect to the underlying layer, a systematic flip of the spins with the field is possible until saturation is reached. The smaller fields to saturate the system in the simulations (~0.2 T) relative to the measurements (~ 1 T) may be due to variations of the magnetic parameters used^36,37^, but the overall picture is well described and in sound agreement with the measurements.

In conclusion, we have shown that twist engineering of magnetic 2D materials is a fruitful platform for the emergence of new correlated phases in artificial metamagnets, as exemplified here by the appearance of multistep spin switching accompanied by hysteretic MR effects in orthogonally twisted bilayer CrSBr. These field-induced features can be controlled by playing with the modulus and direction of the applied magnetic field, which are absent in pristine CrSBr mono- and bilayers. Overall, our results pinpoint twisted bilayer CrSBr as a versatile and rich platform for controlling and addressing the magnetic information on 2D magnets—of special relevance in areas such as spintronics or magnonics^38^—as well as for motivating a new playground for fundamental studies. In particular, this orthogonally twisted bilayer CrSBr may offer a promising route for the creation and manipulation of non-colinear magnetic textures, such as vortices or topologically protected skyrmions and merons^19,20^. In addition, the controlled stacking of 2D magnetic monolayers under defined angles opens new avenues to increase the magnetic symmetry in the plane, thereby reducing the anisotropy energy. Of special interest is to reach the crossover from easy-axis to easy-plane anisotropy, as easy-plane (*XY*) systems^39^ are predicted to host dissipationless spin transport^40,41^.

## Methods

### Crystal growth

CrSBr crystals were synthesized by chemical vapour transport and characterized by powder and crystal X-ray diffraction, energy dispersive X-ray analysis, high-resolution transmission electron microscopy, superconducting quantum interference device magnetometry and temperature-dependent single crystal diffracction, as reported in our previous work^9^.

### vdW heterostructure fabrication

Two-dimensional layers were obtained by mechanical exfoliation from their bulk counterparts under strict inert conditions (argon glovebox) as CrSBr monolayers degrade in air^12,26^. The obtained flakes were examined by optical microscopy (NIKON Eclipse LV-100 optical microscope under normal incidence) as a fast tool for identifying the number of layers, and compared with our previously calibrated values^9^. Typical CrSBr flakes have a ribbon shape, with the long (short) direction associated with the *a* (*b*) axis and the *c* axis in the out-of-plane direction, as verified by optical contrast, Raman spectroscopy and selected area electron diffraction patterns. Details are reported in our previous work^9^. The vdW heterostructures were fabricated by assembling the different layers by deterministic assembly of the flakes using polycarbonate and with the help of a micromanipulator. Thus, the twisted monolayers were placed between top and bottom few-layer metallic NbSe_2_ or few-layer graphene, where several insulating hexagonal boron nitride layers were inserted both to avoid possible shortcuts and to protect the whole heterostructure from degradation. The stack of 2D materials was placed on top of pre-lithographed electrodes (5 nm Ti/50 nm Au on 285 nm SiO_2_/Si from NOVA Electronic Materials). The whole process was performed under inert atmosphere conditions.

A total of three orthogonally twisted CrSBr bilayers were fabricated (device 1 (data shown in the main text) is based on metallic NbSe_2_ thin layers whereas devices 2 and 3 (data shown in Supplementary Figs. 1–9) are based on few-layer graphene), and consistent phenomenology was observed between all of them. Note that in the case of using few-layer graphene, intrinsic MR arising from the few-layer graphene is observed as well (in particular, for out-of-plane applied magnetic fields), yielding a finite positive value of the MR even at room temperature. Nonetheless, the magnetic fingerprints of the twisted CrSBr are well noticeable, clearly developing below *T*_N_.

In particular, device 1 is formed by a top (bottom) CrSBr monolayer of 77.2 µm^2^ (53.3 µm^2^), with an overlap area of 9.3 µm^2^ and a twist angle of 92.5°. Device 2 is formed by a top (bottom) CrSBr monolayer of 190.1 µm^2^ (117.3 µm^2^), with an overlap area of 15.9 µm^2^ and a twist angle of 89.3°. Device 3 is formed by a top (bottom) CrSBr monolayer of 206.6 µm^2^ (121.1 µm^2^), with an overlap area of 7.9 µm^2^ and a twist angle of 87.0°.

### Magnetotransport measurements

Electrical measurements were performed in a Quantum Design PPMS-9 cryostat with a four-probe geometry, where a d.c. current was passed by the outer leads and the d.c. voltage drop was measured in the inner ones. The d.c. voltages and d.c. currents were measured (MFLI from Zurich Instruments) using an external resistance of 1 MΩ, that is, a resistance much larger than that of the sample. Temperature sweeps were performed at 1 K min^−1^, field sweeps at 200 Oe s^−1^, rotation sweeps at 3° s^−1^ and the current bias was 1 µA, unless otherwise explicitly specified. MR is defined as MR = 100[*R*(*B*) – *R*(0)]/*R*(0), where *B* is the external magnetic field and *R*(0) is the resistance at zero field in the symmetric case (see text).

### Atomistic spin dynamic simulations

Our simulations were performed using atomistic spin dynamics simulation techniques^31–34,42–46^ as implemented in the VAMPIRE software package^46^. The energetics of the system is described by the spin Hamiltonian:

$$\begin{array}{c} {\mathcal H} =-\sum _{i < j}{{\bf{S}}}_{i}^{\alpha }{J}_{ij}^{\alpha \beta }{{\bf{S}}}_{j}^{\beta }+\sum _{i < j}{{\bf{D}}}_{ij}\cdot ({{\bf{S}}}_{i}\times {{\bf{S}}}_{j})-{k}_{a}\sum _{i}{({{\bf{S}}}_{i}\cdot \hat{{\bf{a}}})}^{2}-{k}_{b}\sum _{i}{({{\bf{S}}}_{i}\cdot \hat{{\bf{b}}})}^{2}\\ \,-\sum _{i}{{\rm{\mu }}}_{{\rm{s}},i}{{\bf{S}}}_{i}\cdot {\bf{B}}+{ {\mathcal H} }_{{\mathscr{D}}}\end{array}$$

where **S**_*i*_ and **S**_*j*_ are unit vectors describing the local spin directions on Cr sites, represented as *i* and *j*, being *α,β* = *a,b,c*. The first input is the symmetric Heisenberg exchange, which is represented by the exchange tensor between Cr sites, *J*_*ij*_. In the particular case of CrSBr, there are seven intralayer exchange terms (*J*_1–7_), occurring between atoms within the same monolayer, and two interlayer terms, *J*_*z*1_ and *J*_*z*2_, taking place from one monolayer to another. The value of the second intralayer nearest-neighbour exchange (*J*_2_) was taken from ref. ^37^ and was used as a reference to define the magnitude of the *J*_*ij*_ elements, which are outlined in Supplementary Table 1. To obtain satisfactory predictions of the critical temperature of CrSBr-based systems, the relative ratios between exchange parameters were taken from ref. ^47^. For the interlayer interactions, *J*_*z*1_ and *J*_*z*2_, we used the values for the unrotated bilayer due to the absence of marked changes in the intermonolayer distances. The distances for *J*_*z*1_ differ by only about 5.44% and the average deviation in the *J*_*z*2_ interactions is only 2.66%. As commented below, variations of these magnitudes do not change the results.

The second term is the anti-symmetric exchange or DMI which stabilizes topological states, where **D**_*ij*_ is the DMI vector. Due to the absence of inversion symmetry between interacting Cr-based atoms^48^, we have included the reported anti-symmetric contributions with DMI unit vectors parallel to the *a*th (mediating *J*_3_) and *b*th (mediating *J*_1_) axes, whose values are given, respectively, by *D*_1_ = 0.07 meV and *D*_3_ = 0.18 meV (ref. ^47^).

The third term is the on-site anisotropy energy, which is made up of two uniaxial terms, where the relative values of the anisotropy constants, *k*_*a*_ = 8.06 meV and *k*_*b*_ = 31.53 meV, govern the intermediate *a*th and easy *b*th axes of the system^37^. It is important to note that previously introduced single-ion anisotropies are not, theoretically, the only ones that should contribute to the overall magnetocrystalline anisotropy of the system. The larger spin–orbit coupling of the Br atoms compared with Cr atoms points to the existence of in-basal-plane-based exchange anisotropy terms at the previously defined spin Hamiltonian^49^. However, in the computational characterization of the system for the twisted bilayer, we have chosen not to include them, despite the fact that it has been reported that they share the same order of magnitude as the on-site anisotropy contributions. This is because these second-ion terms can induce the *a*th axis to be the easiest one in the system^37^. Moreover, the single-ion contributions are enough to unravel the main features observed experimentally. It is worth noting that, due to the rotation process, the easy axis of the top monolayer is directed along the *a*th spatial direction and the intermediate one the *b*th axis (orthogonally directed with respect to the untwisted bottom monolayer).

The fifth term is the Zeeman energy, where **B** represents the externally applied magnetic field and *μ*_s_ is the atomic magnetic moment, to which the value *μ*_s_ = 2.88*μ*_B_ has been assigned in consonance with the bulk scenario^36^, with *μ*_B_ being the Bohr magneton.

The final term is the long-range dipole–dipole interaction, ${ {\mathcal H} }_{{\mathscr{D}}}$, which can be expressed as:

$${ {\mathcal H} }_{{\mathscr{D}}}=\frac{{{\rm{\mu }}}_{0}{\mu }_{{\rm{s}}}^{2}}{4{{\uppi }}}\sum _{j}\left[\frac{3{\hat{{\bf{r}}}}_{ij}({\hat{{\bf{r}}}}_{ij}\cdot {{\bf{S}}}_{j})-{{\bf{S}}}_{j}}{{|{{\bf{r}}}_{ij}|}^{3}}\right]$$

where |**r**_*ij*_| is the distance between site *i* and site *j*.

We also calculated the interlayer exchange field as:

$${{\bf{H}}}_{{\rm{exc}}}^{{\rm{inter}}}=\frac{1}{{\mu }_{{\rm{s}}}}[-4|{J}_{z1}|({{\bf{m}}}_{{\rm{bottom}}}+{{\bf{m}}}_{{\rm{top}}})+{J}_{z2}({m}_{{\rm{bottom}}}^{c}+{m}_{{\rm{top}}}^{c})\hat{{\bf{c}}}]$$

where the magnetization of the bottom and top layer is represented by **m**_bottom_ and **m**_top_, respectively, and **ĉ** is a unit vector along the out-of-plane direction. Taking into account that there are four nearest neighbours, mediated by *J*_*z*1_, and one next-nearest neighbour, mediated by *J*_*z*2_, interactions, we can estimate a maximum exchange field of ~0.15 T if we assume that **m**_bottom_ and **m**_top_ are fully parallel. This magnitude is much smaller than the dipolar fields induced by the twisted layer (Supplementary Fig. 14), and suggests that variations of the order of 5–10% in exchange interactions will not affect the results in case the rotation might play a role. This correlates with potential variations due to the interlayer distance between Cr sites as commented above.

## Online content

Any methods, additional references, Nature Portfolio reporting summaries, source data, extended data, supplementary information, acknowledgements, peer review information; details of author contributions and competing interests; and statements of data and code availability are available at 10.1038/s41563-023-01735-6.

### Supplementary information

Supplementary Information Supplementary Figs. 1–16, caption for Videos 1–7 and Table 1.

Supplementary Video 1 Multistep magnetization switching with magnetic memory in orthogonally twisted bilayer CrSBr as shown in Fig. 3

Supplementary Video 2 Video of the spin dynamic simulation at field cooling of 0.06 mT from above 200 K towards 0 K. The magnetic field is applied following the schematic in the inset of Fig. 4a with the field parallel to the easy axis of the bottom layer. The simulation time comprises 2 ns of the cooling process. An additional 1.5 ns simulation time is undertaken at 0 K to check further the stability The colour scheme follows that in Supplementary Fig. 11.

Supplementary Video 3 Similar to Supplementary Video 2 at an applied field of 0.10 T. The formation of domain walls occurred at the top layer as it is still rotating to align with the field.

Supplementary Video 4 Similar to Supplementary Video 2 at an applied field of 0.20 T.

Supplementary Video 5 Similar to Supplementary Video 2 with a field of 0.06 mT oriented antiparallel to the easy axis of the bottom layer (−*B*_*x*_).

Supplementary Video 6 Similar to Supplementary Video 5 with a field of *B*_*x*_ = −0.12 mT.

Supplementary Video 7 Similar to Supplementary Video 4 with a field of *B*_*x*_ = −0.2 mT. Note that in general, the formation of the domain walls, spin textures and so on occurred at the top layer (easy axis perpendicular to the field) despite the direction of the applied field (±*B*_*x*_). When the field is reversed (−*B*_*x*_), however, the magnetic structure of the bottom layer (antiparallel to the field) becomes more inhomogeneous with more fluctuations of the spins

## Data Availability

The dataset that supports the findings of this study is available via the 4TU.ResearchData repository at 10.4121/3ae7c6fa-879c-4dcd-a515-11c13151d7b0.

## Code availability

The code that supports the findings of this study is available via the 4TU.ResearchData repository at 10.4121/3ae7c6fa-879c-4dcd-a515-11c13151d7b0.
