# Supplementary material for: Multistep magnetization switching in orthogonally twisted ferromagnetic monolayers
Source: Nat Mater. 2023 Nov 30;23(2):212–8. doi: 10.1038/s41563-023-01735-6 (PMC10837074; doi:10.1038/s41563-023-01735-6)
Supplement: Supplementary file 1 — Supplementary Figs. 1–16, caption for Videos 1–7 and Table 1. [file 41563_2023_1735_MOESM1_ESM.pdf]

nature materials

Article https://doi.org/10.1038/s41563-023-01735-6

Multistep magnetization switching in orthogonally twisted ferromagnetic monolayers

In the format provided by the authors and unedited

Table of contents

Section A – Supplementary Figures 1 - 16 .... S2
Section B – Supplementary Movies 1 – 7 .... S16
Section C – Supplementary Table I ……………………………………………………….S16

Section A - Supplementary Figures 1 - 16

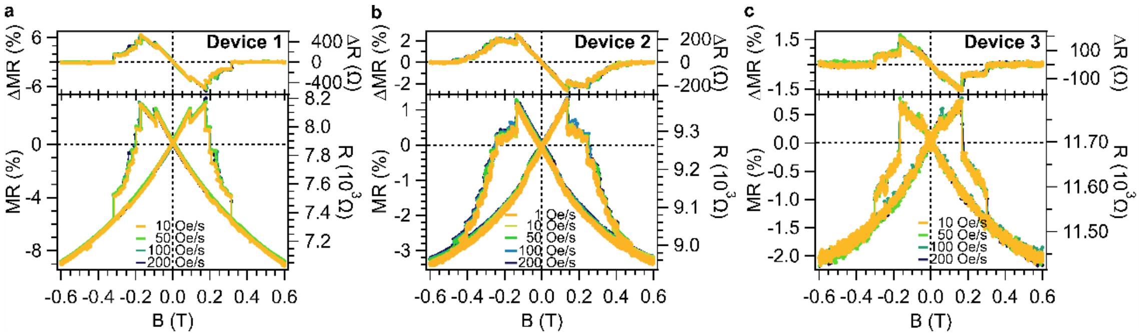

Supplementary Figure 1.- Magnetic field dependence of the magneto-resistance (MR) in orthogonally-twisted bilayer CrSBr for different devices and field sweep rates. a, Device based on $NbSe_2$ vertical van der Waals heterostructure (T = 10 K). **b-c,** Devices based on few-layers graphene vertical van der Waals heterostructures (T = 2 K). The field is applied in-plane (β = 0º) along the easy-axis of the CrSBr monolayer with smaller area, corresponding to α = 0º (for **a** and **b**) and α = 90º (for **c**). MR is defined as MR (%) = 100·[R(B) – R(0)]/R(0), being R(0) the resistance obtained at zero field.

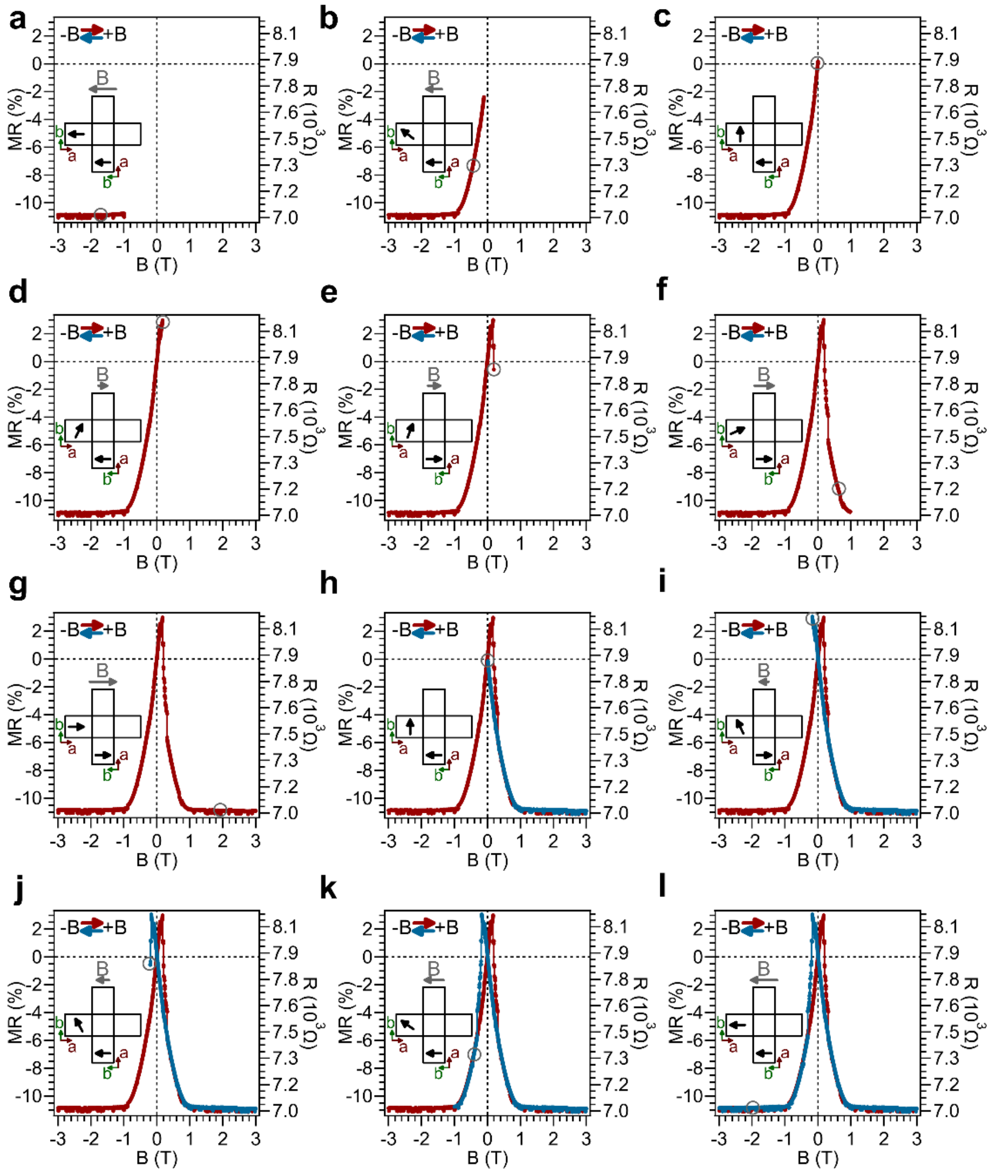

Supplementary Figure 2.- Magnetization switching for an orthogonally-twisted bilayer CrSBr. The applied external magnetic field (represented as grey arrow and position marked as grey circle) is aligned with the easy-axis (*b*) of one of the monolayers and aligned with the intermediate-axis (*a*) of the *rotated* monolayer. The magnetization for every layer is represented as a black arrow. **a,** At high negative magnetic fields, the magnetization of both layers is parallel and aligned with the field and, therefore, the resistance is minimum within a spin-valve model. **b,** Below -1 T, the magnetization of the *rotated* monolayer starts canting towards its easy-axis. The magnetization of both layers is not parallel, yielding to an increase of the resistance. **c,** At zero field, the magnetization of both layers is orthogonal assuming negligible inter-layer interactions. **d,** At small positive magnetic fields, the magnetization of the *rotated* monolayer starts canting towards the direction of the magnetic field. The angle between the magnetization of the two layers increases towards an antiparallel state and, therefore, the resistance increases. **e,** At *ca.* 0.16 T, the monolayer with its easy-axis along the field flips its magnetization (spin flip). Thus, the angle between the magnetization of both layers suddenly decreases and, then, the resistance drops. **f,** Applying higher positive magnetic fields cants the magnetization of the *rotated* monolayer towards the direction of the magnetic field. The angle between the magnetization of both layers decreases and, therefore, the resistance diminishes. **g,** For fields above 1 T, the magnetization of both layers is parallel along the direction of the applied field and the resistance is minimum. **h,** For fields below 1 T, the magnetization of the *rotated* layer starts canting towards its easy axis, yielding to a resistance increase. At zero field, the magnetization of both layers is orthogonal assuming negligible inter-layer interactions. **i,** At small negative magnetic fields, the magnetization of the *rotated* monolayer starts canting towards the direction of the magnetic field. The angle between the magnetization of the two layers increases towards an antiparallel state and, therefore, the resistance increases. **j,** At *ca.* -0.16 T, the monolayer with its easy-axis along the field flips its magnetization (spin flip). Thus, the angle between the magnetization of both layers suddenly decreases and, then, the resistance drops. **k,** Applying higher negative magnetic fields cants the magnetization of the *rotated* monolayer towards the direction of the magnetic field. The angle between the magnetization of both layers decreases and, therefore, the resistance diminishes. **l,** For fields below -1 T, the magnetization of both layers is parallel along the direction of the applied field and the resistance is minimum.

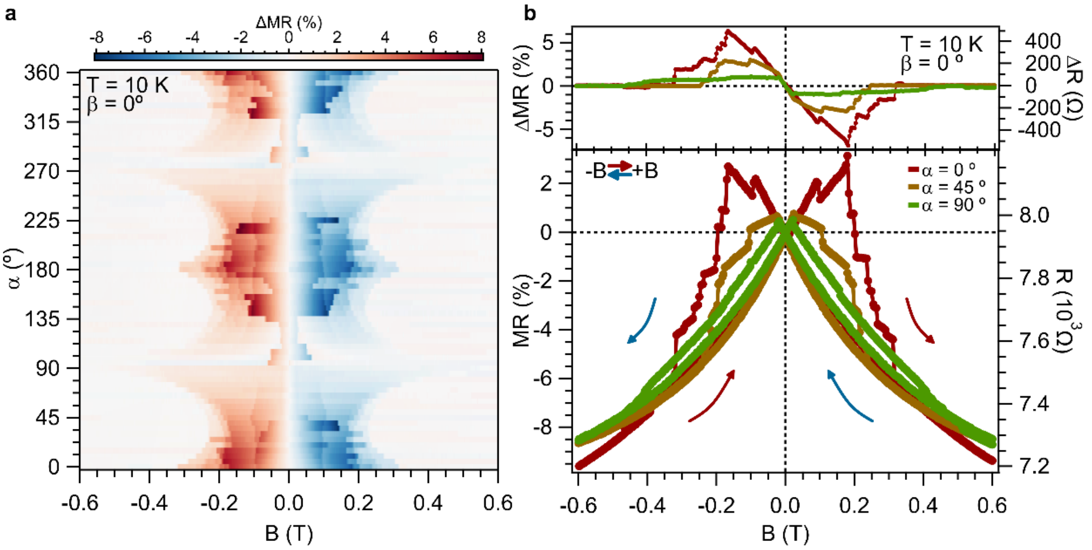

Supplementary Figure 3.- In-plane magnetic field dependence of the magneto-resistance (MR) in orthogonally-twisted bilayer CrSBr (device 1). a, 2D plot of ΔMR. **b,** Selected MR/resistance hysteresis loops (bottom panel) and its increment (top panel) at selected angles. Measurements corresponds to an orthogonally-twisted CrSBr bilayer based on metallic $NbSe_2$ thin-layers vertical van der Waals heterostructure. MR is defined as MR (%) = 100·[R(B) – R(0)]/R(0), being R(0) the resistance obtained at zero field.

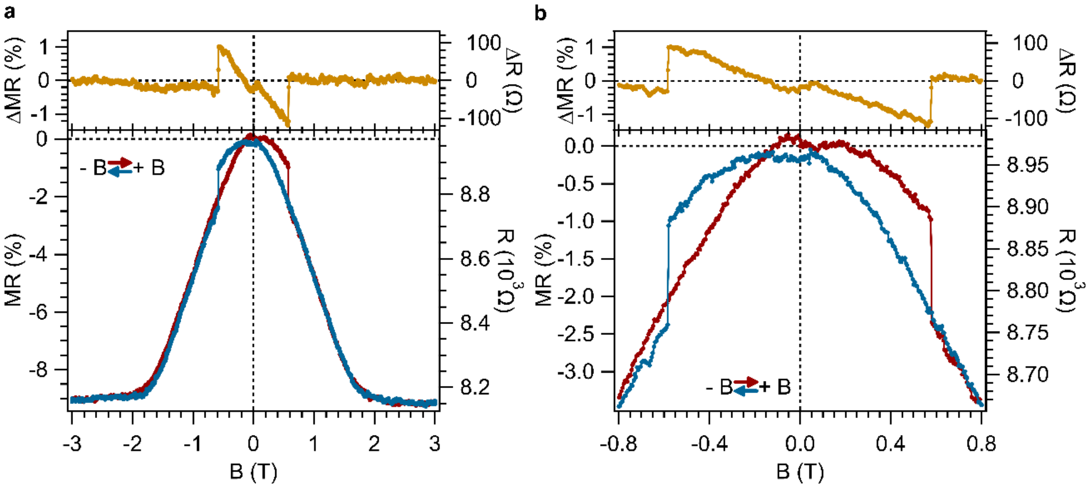

Supplementary Figure 4.- Out-of-plane magnetic field dependence of the magneto-resistance (MR) in orthogonally-twisted bilayer CrSBr (device 1). a,b, Field-dependence of the resistance and MR (bottom panel) as well as its increment (top panel), defined as $\Delta X = X_{+B\to-B} - X_{-B\to+B}$, where X states either for the resistance or the MR (T = 10 K, α = 0º and β = 90º). Sweeping up (down) trace is depicted in red (blue).

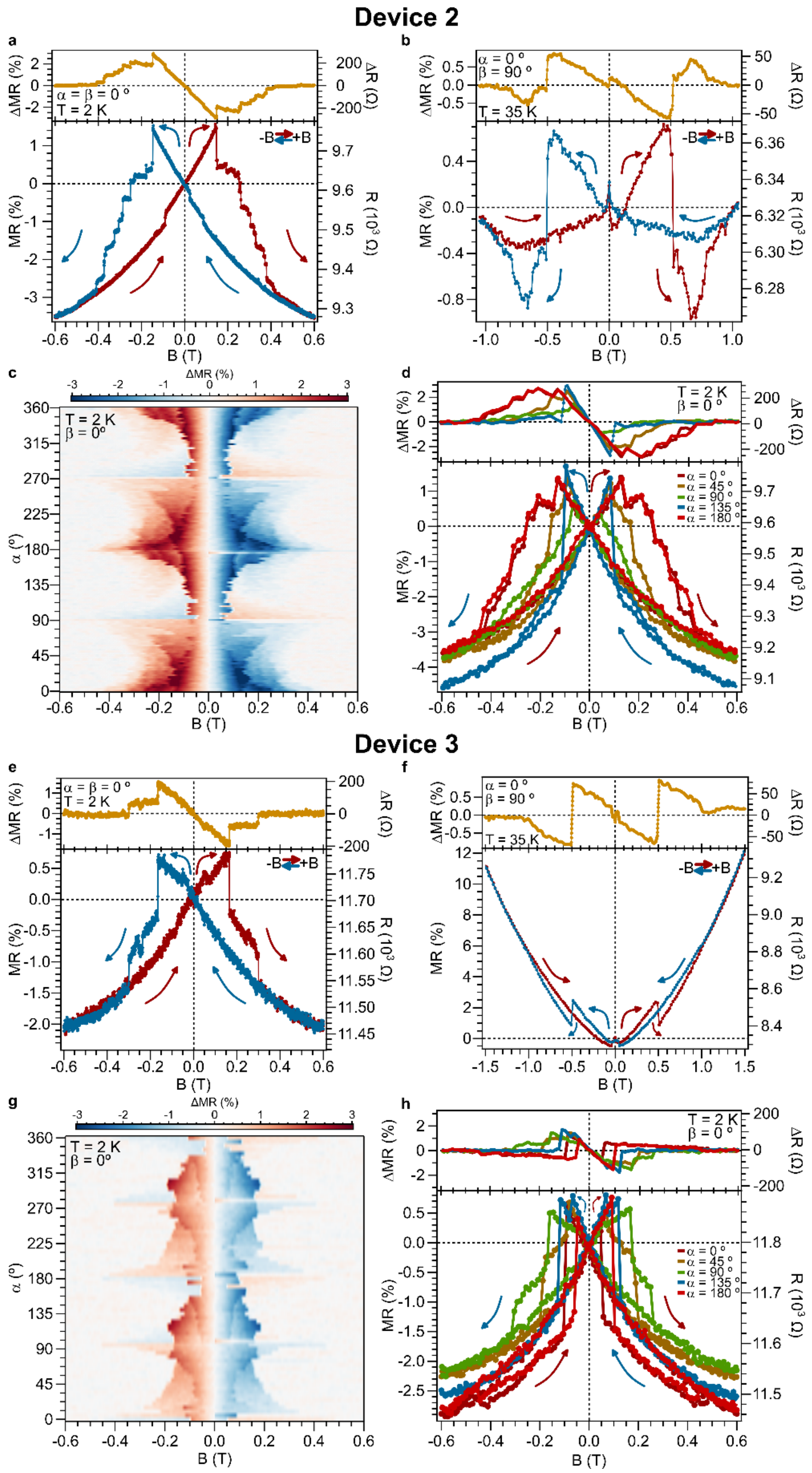

Supplementary Figure 5.- Magnetic field dependence of the magneto-resistance (MR) in orthogonally-twisted bilayer CrSBr based on few-layers graphene van der Waals heterostructures. Panels **a-d** (**e-h**) correspond to device 2 (3). **a,b,e,f,** Field-dependence of the resistance and MR (bottom panel) as well as its increment (top panel), defined as $\Delta X = X_{+B\rightarrow-B} - X_{-B\rightarrow+B}$, where X states either for the resistance or the MR for in-plane (**a,e** panels) and out-of-plane (**b,f** panels) fields. Sweeping up (down) trace is depicted in red (blue). Red/blue arrows indicate the sweeping direction of the magnetic field. MR is defined as MR (%) = 100·[R(B) – R(0)]/R(0). **c,g,** 2D plot of ΔMR. **d,h,** Selected MR and resistance hysteresis loops (bottom panel) and its increment (top panel) at selected angles. Note that the intrinsic MR arising from the few-layers graphene is observed as well (in special, for out-of-plane applied magnetic fields), yielding to a finite positive value of the MR even at room temperature. Nonetheless, the magnetic fingerprints of the twisted-CrSBr are well noticeable.

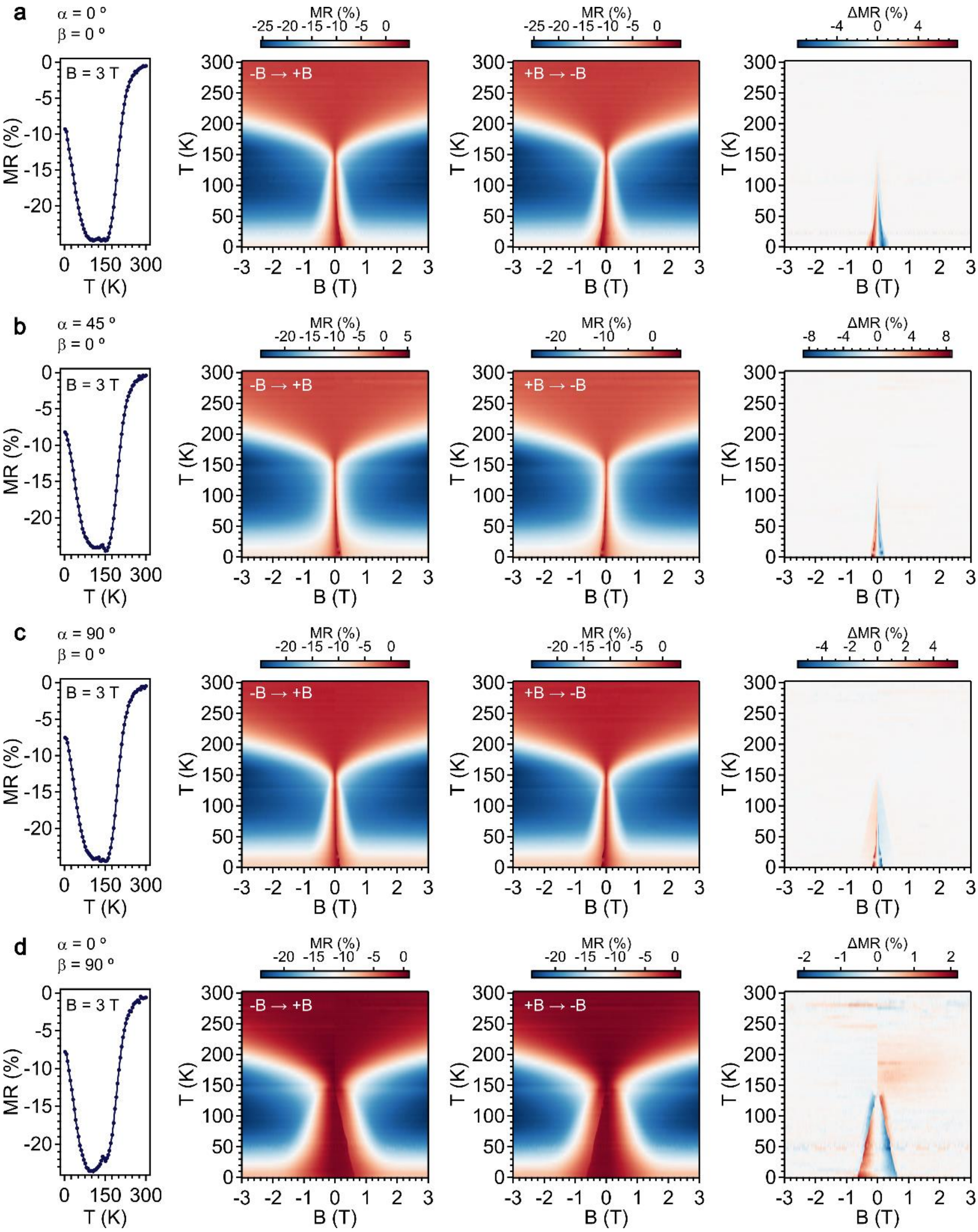

Supplementary Figure 6.- Temperature and magnetic field dependence in orthogonally-twisted bilayer CrSBr. a-c, In-plane field orientations. **d,** Out-of-plane orientation. First panel: Temperature dependence of the magneto-resistance (MR) in the saturated state (B = 3 T). Second (third) panel: field and temperature dependence of the MR while sweeping from negative (positive) to positive (negative) fields. Fourth panel: field and temperature dependence of ΔMR. MR is defined as MR (%) = 100·[R(B) – R(0)]/R(0), being R(0) the resistance obtained at zero field.

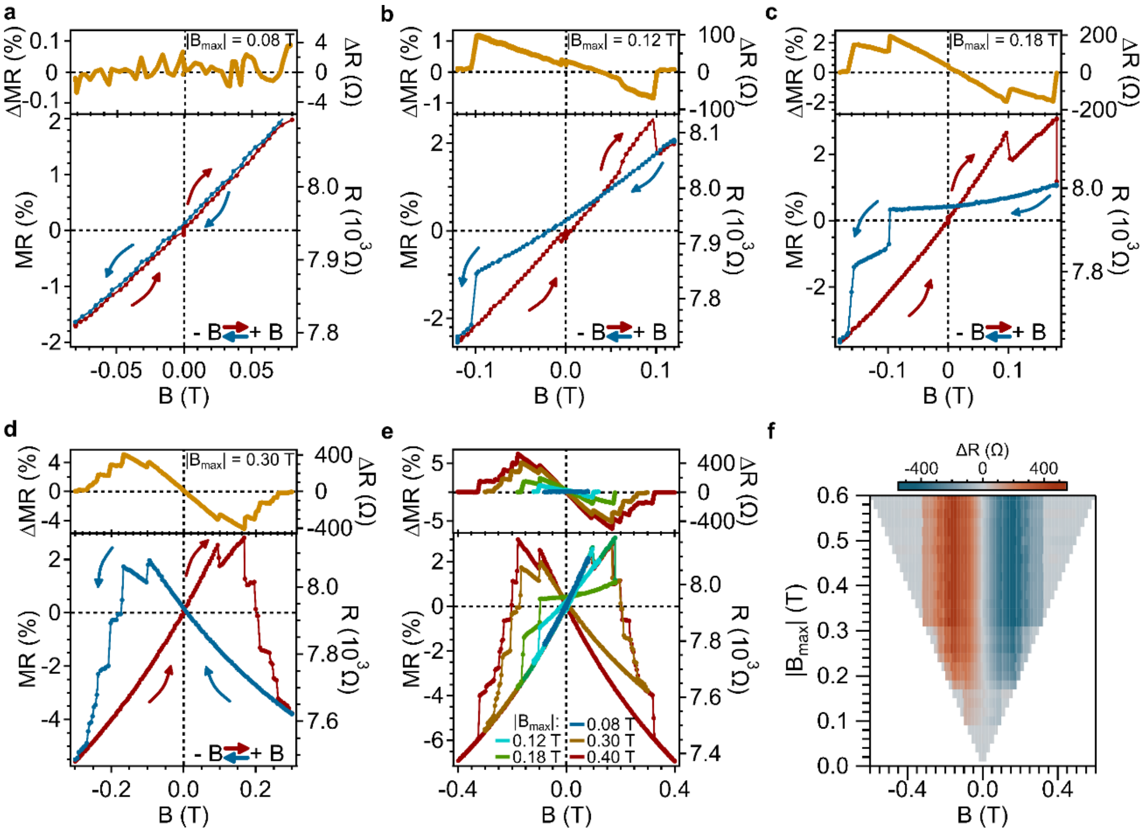

Supplementary Figure 7.- Hysteresis opening in orthogonally-twisted bilayer CrSBr (device 1). a-e, Field-dependence of the resistance and magneto-resistance (MR) (bottom panel) as well as its increment (top panel), defined as $\Delta X = X_{+B\rightarrow -B} - X_{-B\rightarrow +B}$, where X states either for the resistance or the MR after sweeping up to different selected magnetic fields at 10 K and $\alpha = \beta = 0°$, being the magnetic field applied in plane along the easy (intermediate) magnetic axis of the top (bottom) CrSBr monolayer. **f,** ΔR 2D plot. The magnetic sweep protocol is as follows: after a first saturation at negative fields, we perform the sequence ZF ⟶ B_{max} ⟶-B_{max}⟶ ZF, increasing in every cycle the maximum field in 20 mT step. Sweeping up (down) trace is depicted in red (blue) in **a-d**. Red/blue arrows indicate the sweeping direction of the magnetic field. MR is defined as MR (%) = 100·[R(B) – R(0)]/R(0), being R(0) the resistance obtained at zero field in the symmetric case.

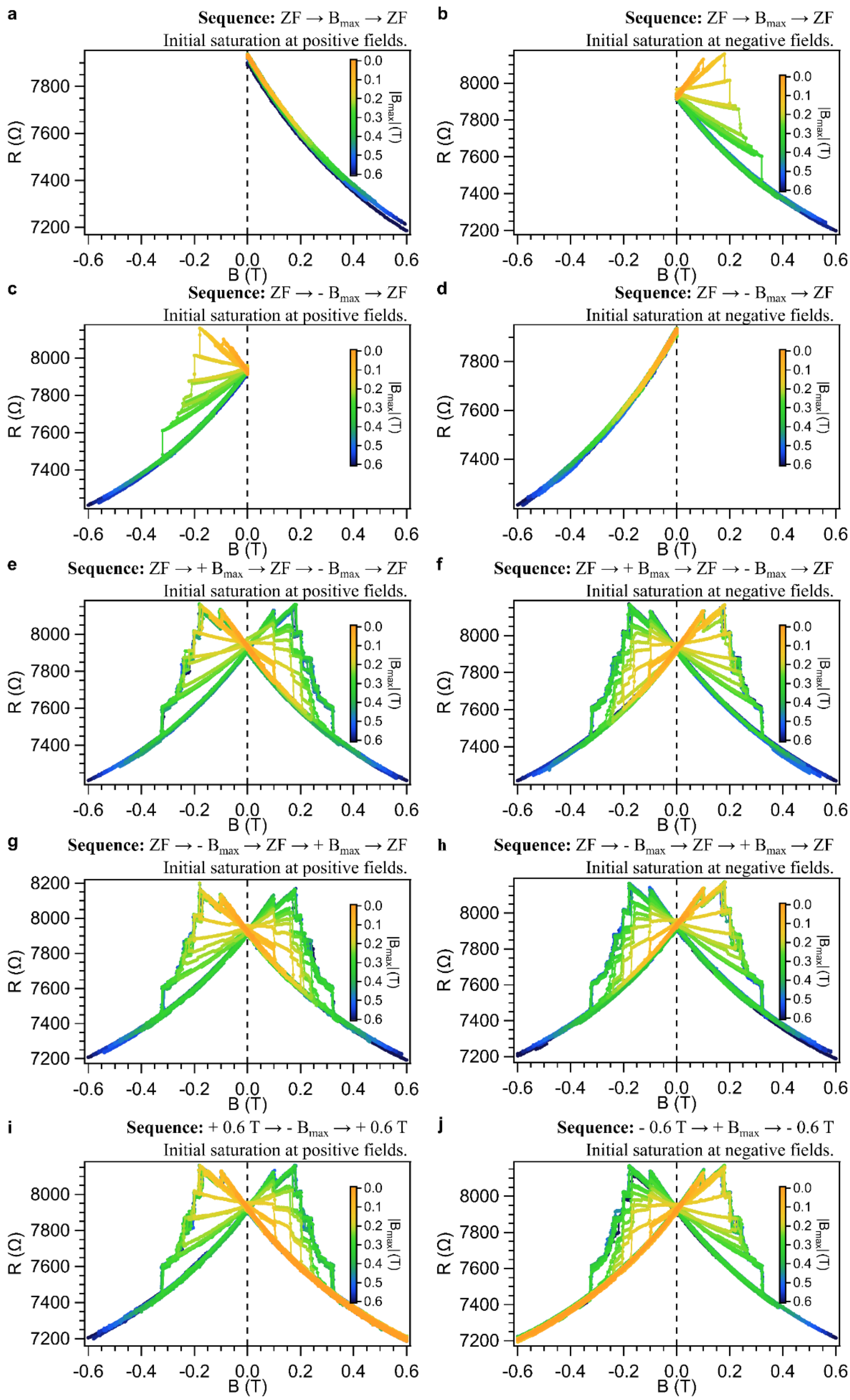

Supplementary Figure 8.- Multistep spin switching with magnetic memory in orthogonally-twisted CrSBr under different magnetic field sweep protocols. T = 10 K and α = β = 0 ° (device 1). **a,b,** Sequence ZF ⟶ +B$_{max}$ ⟶ ZF. **c,d,** Sequence ZF ⟶ -B$_{max}$ ⟶ ZF. **e,f,** Sequence ZF ⟶ +B$_{max}$ ⟶ ZF ⟶ -B$_{max}$ ⟶ ZF. **g,h,** Sequence ZF ⟶ -B$_{max}$ ⟶ ZF ⟶ +B$_{max}$ ⟶ ZF. **i,** Sequence +0.6 T ⟶ -B$_{max}$ ⟶ + 0.6 T. **j** Sequence -0.6 T ⟶ +B$_{max}$ ⟶ - 0.6 T. Panels **a**, **c**, **e**, **g** and **i** (**b**, **d**, **f**, **h** and **j**) correspond to an initial saturation at positive (negative) magnetic fields. In every field sweep, B$_{max}$ is incremented in steps of 20 mT.

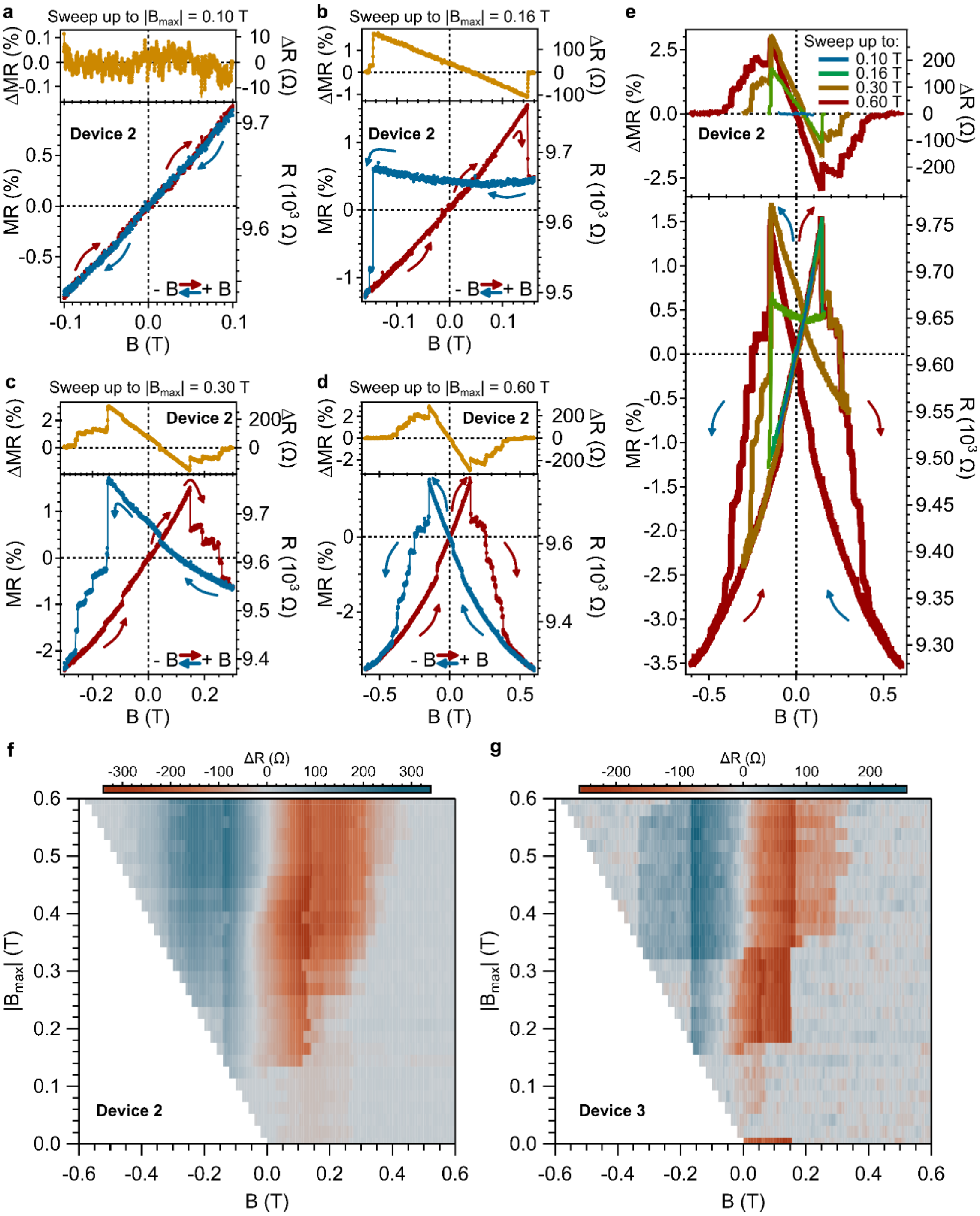

Supplementary Figure 9.- Hysteresis opening in orthogonally-twisted bilayer CrSBr based on few-layers graphene vertical van der Waals heterostructures. a-e, Field-dependence of the resistance and magneto-resistance (MR) (bottom panel) as well as its increment (top panel), defined as $\Delta X = X_{+B\rightarrow-B} - X_{-B\rightarrow+B}$, where X states either for the resistance or the MR after sweeping up to different selected magnetic fields at 2 K and $\alpha = \beta = 0$ ° (device 2). **f-g,** ΔR 2D plot. The magnetic sweep protocol is as follows: for panels **a-e**, after a first saturation at negative fields, we perform the sequence ZF → B_{max} →-B_{max}→ ZF, increasing in every cycle the maximum field in 20 mT step. In panel **f-g**, the sequence is +0.6 T → -B_{max} →+0.6 and increasing in every cycle the maximum negative field in 20 mT step, for device 2 (**f**) and device 3 (**g**). Sweeping up (down) trace is depicted in red (blue) in **a-d**. Arrows indicate the sweeping direction of the magnetic field. MR is defined as MR (%) = 100·[R(B) – R(0)]/R(0), being R(0) the resistance obtained at zero field in the symmetric case.

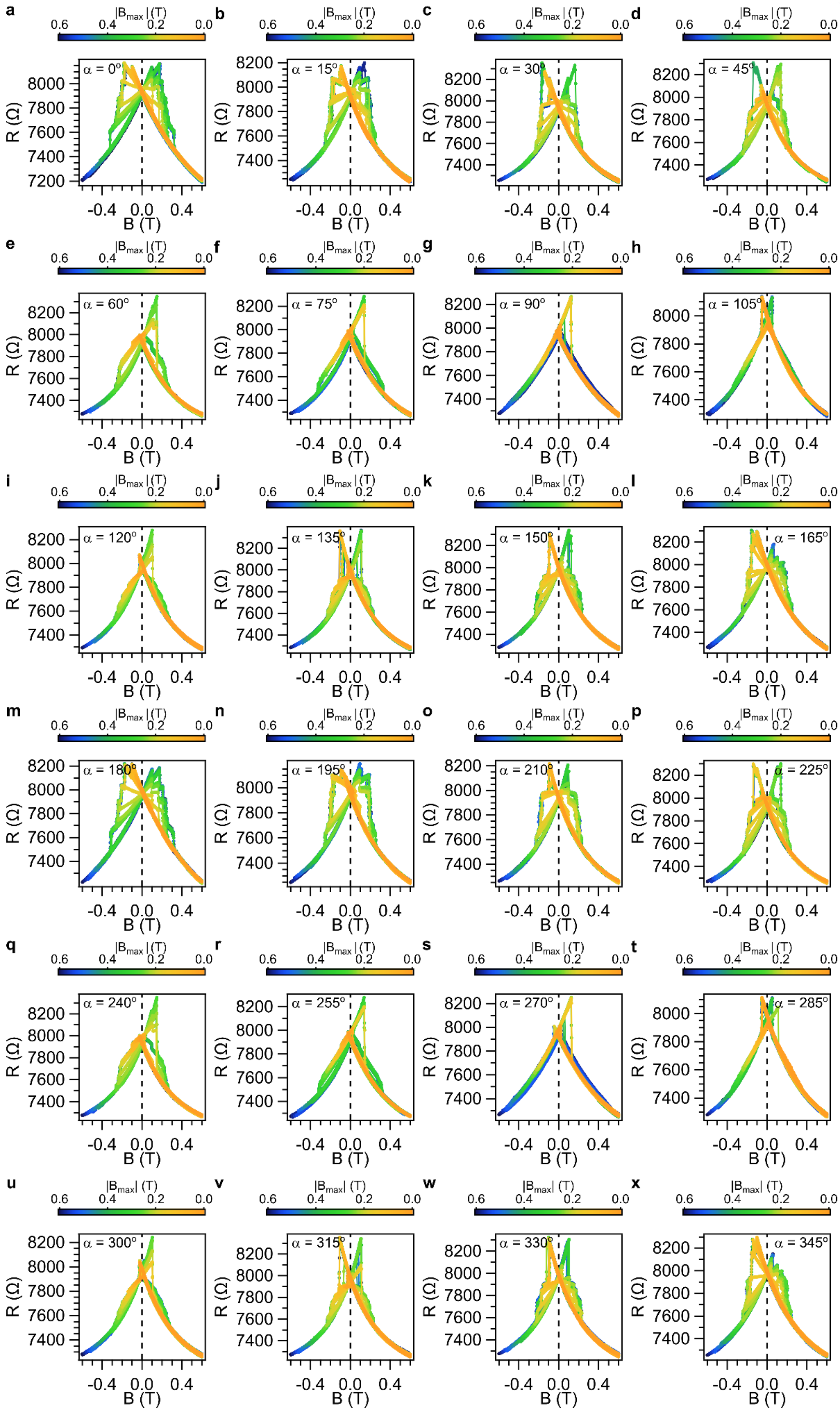

Supplementary Figure 10.- First-order reversal curves for different in-plane (β = 0 °) magnetic fields at T = 10 K (device 1). We consider the sequence +0.6 T → $-B_{max}$ → + 0.6 T. In every field sweep, B_{max} is incremented in steps of 20 mT.

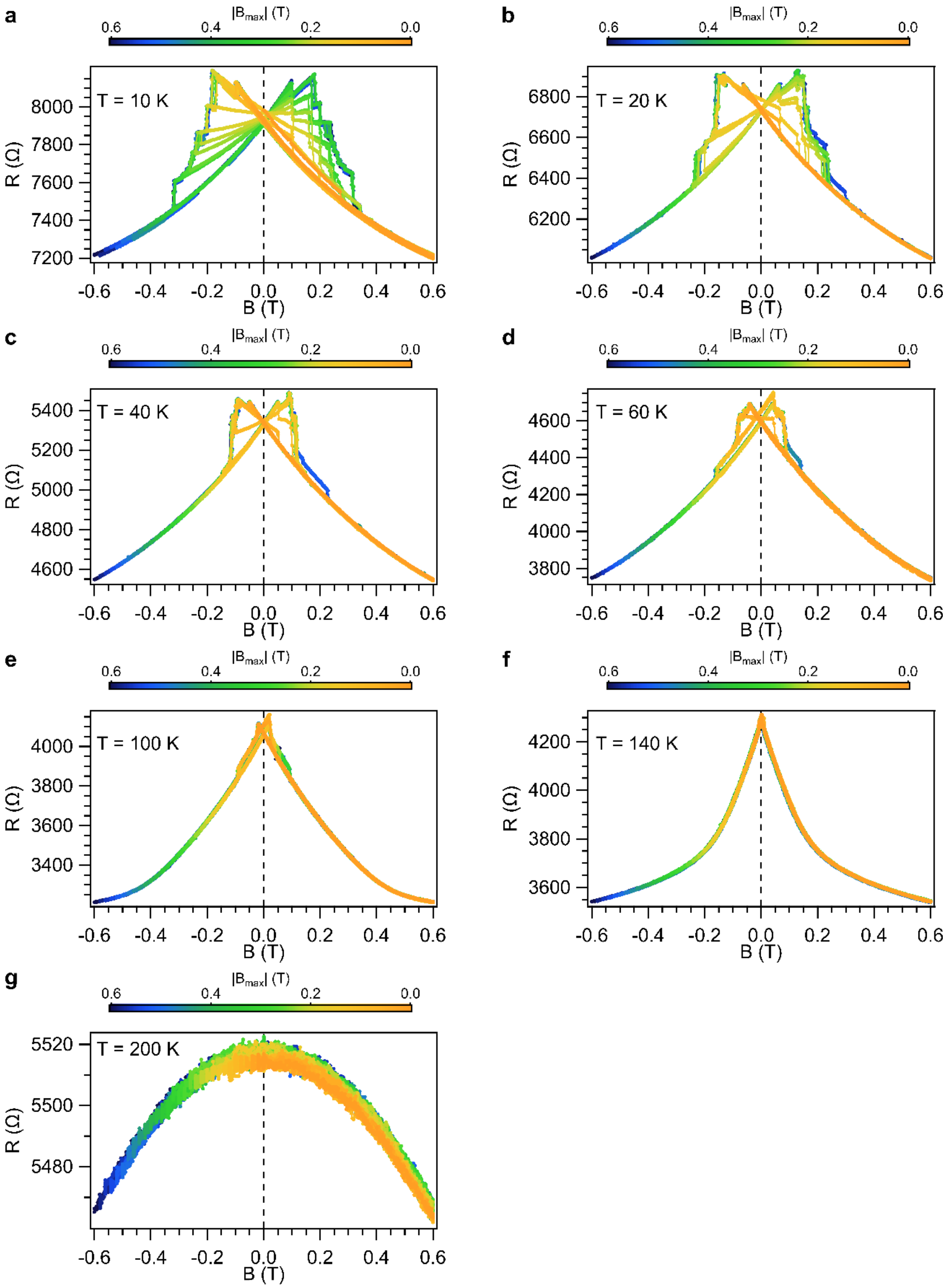

Supplementary Figure 11.- First-order reversal curves for in-plane (α = β = 0 °) magnetic fields at different temperatures (device 1). We consider the sequence +0.6 T → $-B_{max}$ → + 0.6 T. In every field sweep, B_{max} is incremented in steps of 20 mT.

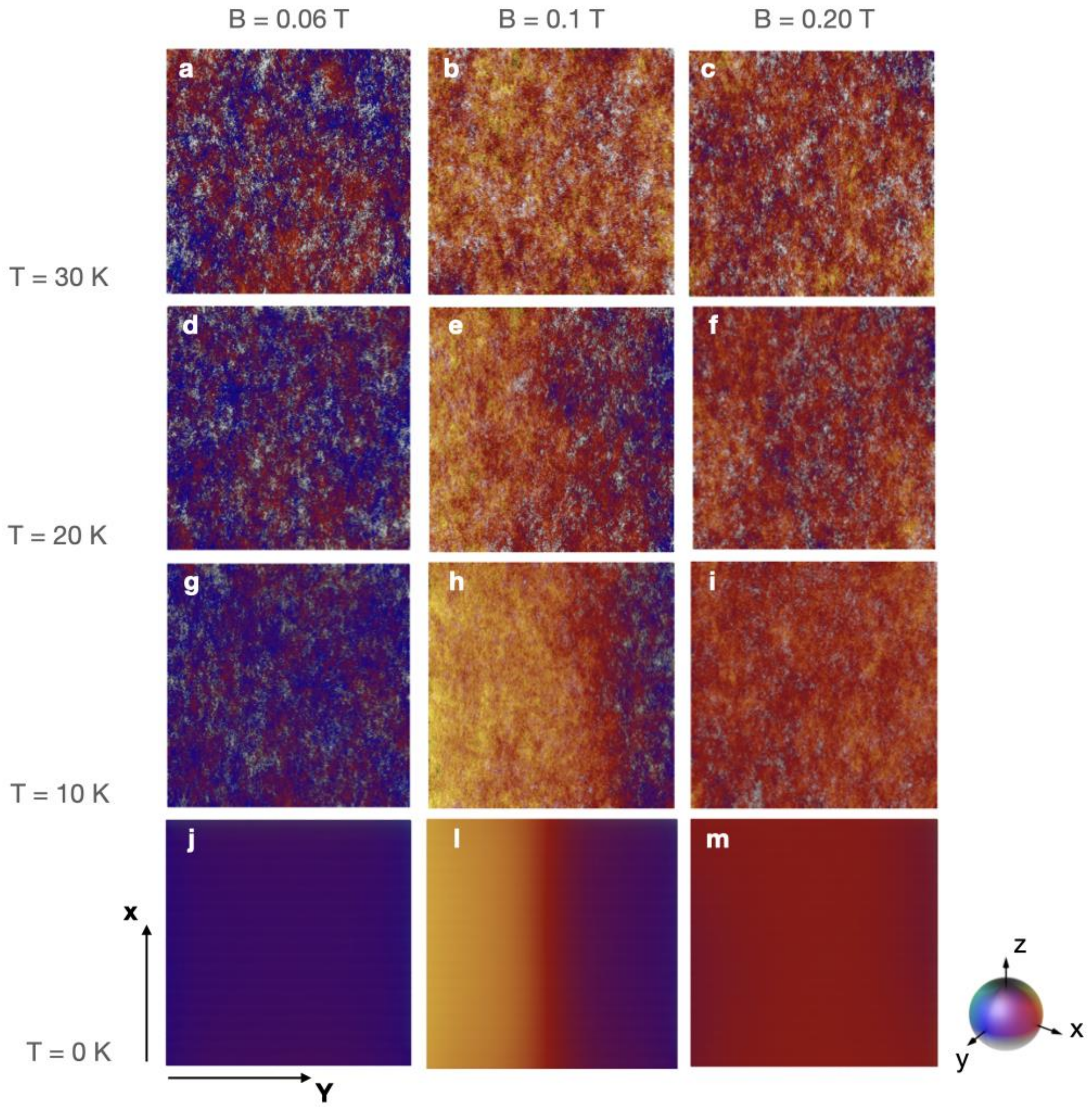

Supplementary Figure 12.- Spin dynamics simulations for orthogonally-twisted bilayer CrSBr. a-m, Snapshots of the spin configurations during cooling at different applied fields (0.06 T, 0.10 T, 0.20 T) and temperatures: 30 K (**a-c**), 20 K (**d-f**), 10 K (**g-i**), 0 K (**j-m**). The field is applied following the configuration displayed in the inset of **Figure 4a**. That is, at zero field the easy-axis at both layers are perpendicular to each other due to the device configuration created. As the field is increased, the magnetization of the layer which initially has its easy-axis perpendicular to field (e.g., top layer) rotates to be aligned with the field. The different spin-textures are formed during this process at the corresponding layer not totally oriented with the field. The other layer (e.g., bottom layer) which has its easy-axis already oriented with the external field does not play a substantial role in the phenomena. The spins are oriented accordingly to the magnetic axes displayed in the inset of **Figure 4a**. Each panel measures 100 nm × 100 nm along *y-th* and *x-th* axis with no periodic boundary conditions. Edges have not observed to play any variation on the results.

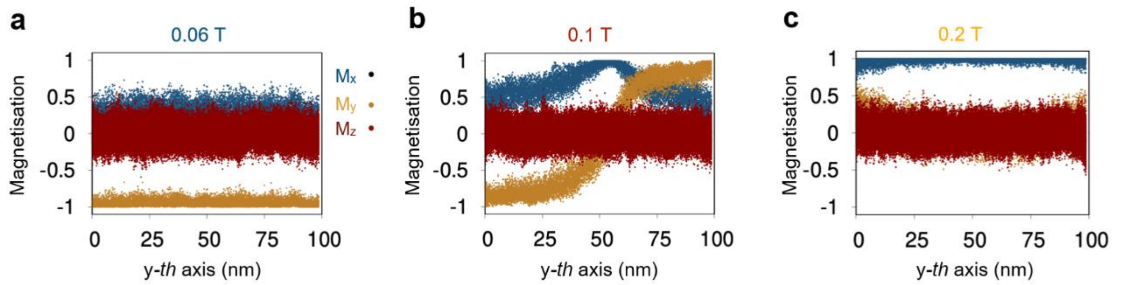

Supplementary Figure 13.- Field-induced spin-textures in orthogonally-twisted bilayer CrSBr a-c, Projections of the magnetisation M_x, M_y and M_z at 5 K as a function of the position (nm) along the *y*-axis at 0.06 T, 0.1 T and 0.2 T, respectively. See schematic in the inset of **Fig. 4a** for field geometry.

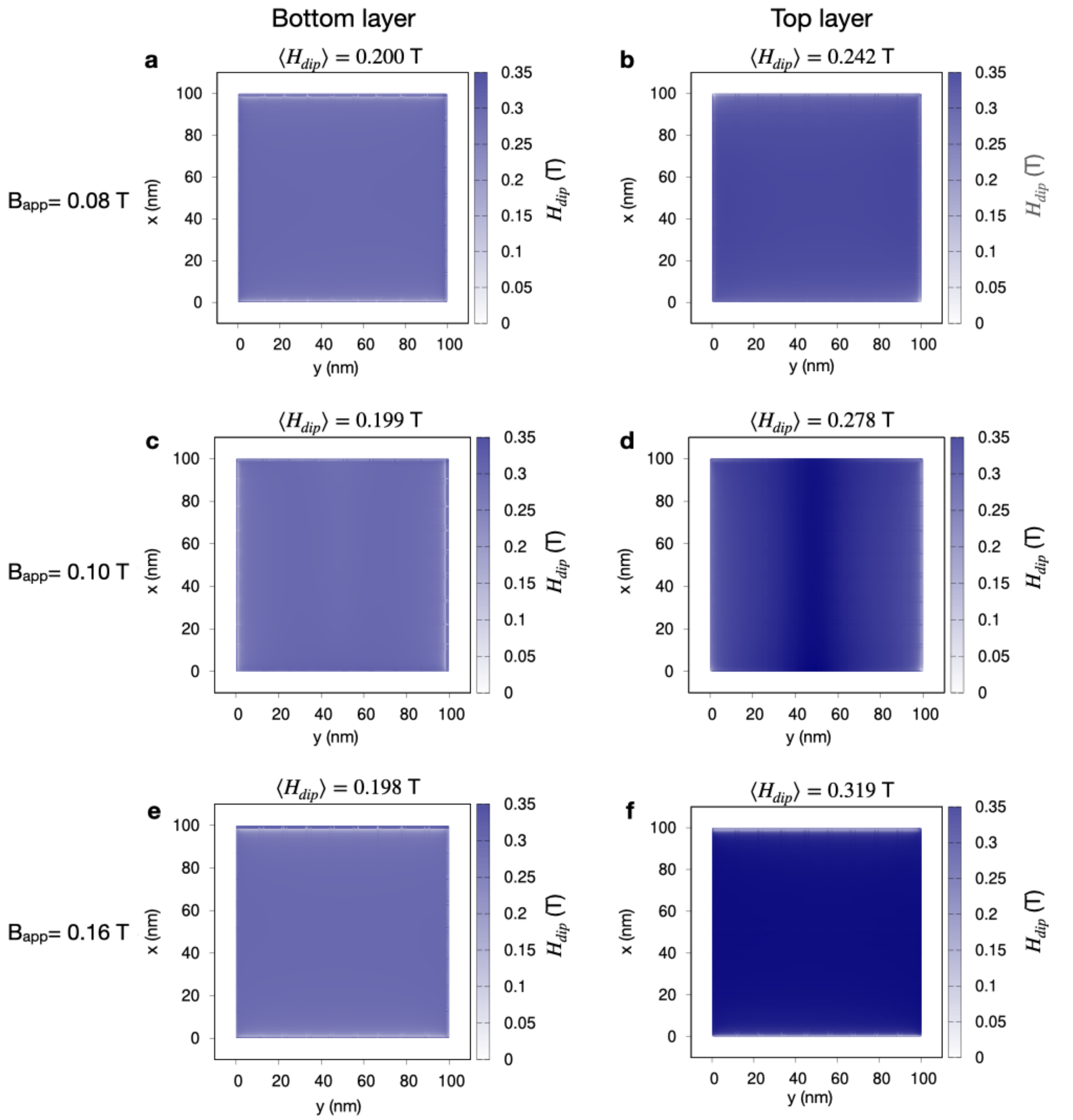

Supplementary Figure 14.- Dipolar field maps extracted for orthogonally-twisted bilayer CrSBr. a-f, Dipolar fields (H_{dip}) projected over bottom and top layers which have easy-axis parallel and perpendicular, respectively, to the applied field (B_{app}). See schematic in the inset of **Fig. 4a** for field geometry. Different magnitudes of B_{app} are applied (0.08 T (**a-b**), 0.10 T (**c-d**) and 0.16 T (**e-f**)), with the corresponding average values of the dipolar fields $\langle H_{dip} \rangle$ induced in the system included at the top of each panel.

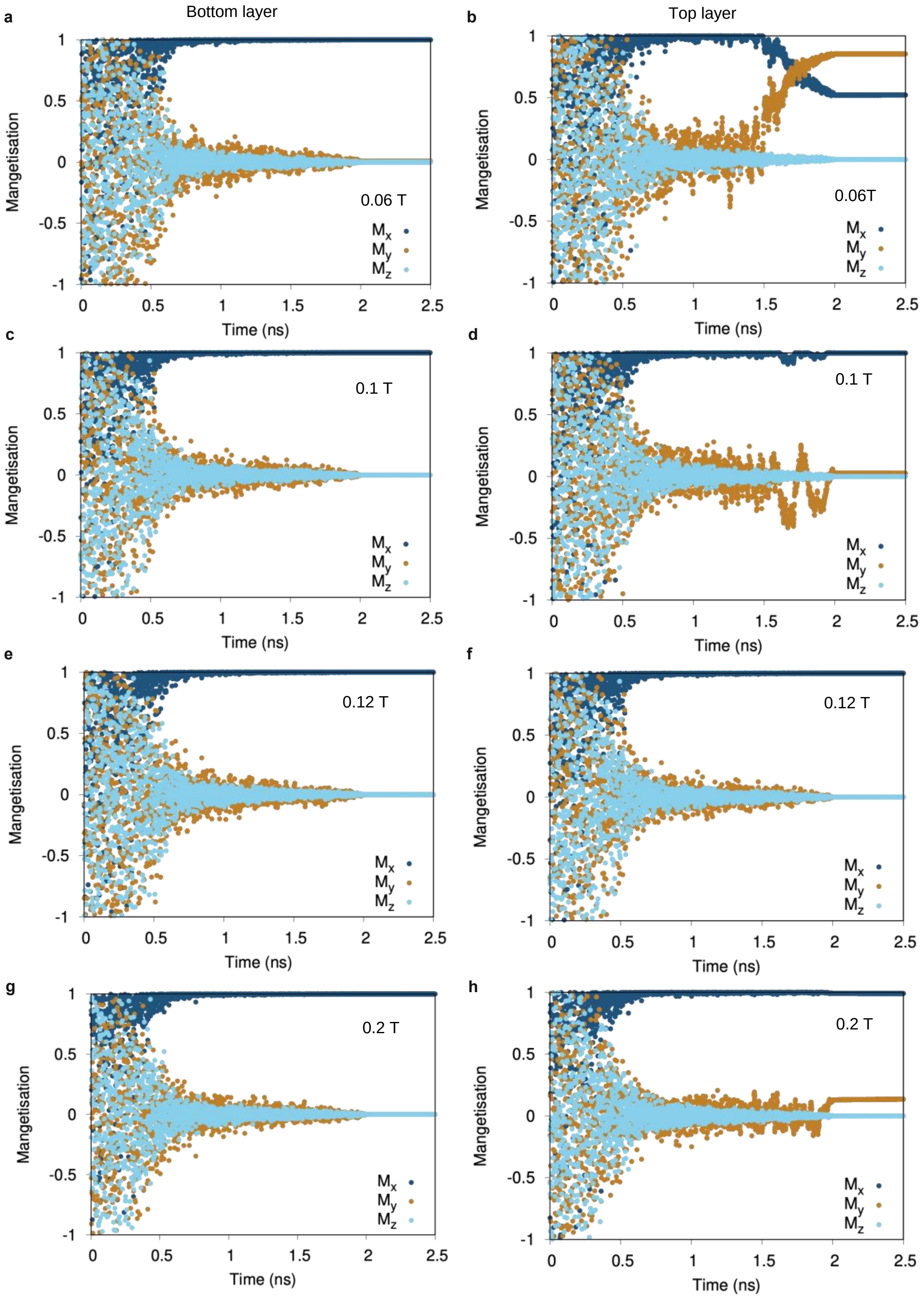

Supplementary Figure 15.- Time variation of the spins at orthogonally-twisted bilayer CrSBr. a-h, Projection of the magnetisation along M_x, M_y and M_z components for the bottom and top layers as function of time. The magnetic field **B** is applied following the inset of **Figure 4a** and it is parallel (B_x) and orthogonal to the easy-axis of the bottom and top layers, respectively. A time interval of 2 ns is recorded for the variations of M_x, M_y and M_z under different fields: 0.06 T (**a-b**), 0.1 T (**c-d**), 0.12 T (**e-f**), 0.2 T (**g-h**). The initial spin configurations at 0 ns are randomly assigned at the beginning of the atomistic spin dynamics which generated the large variations of M_x, M_y and M_z with time until convergence is achieved.

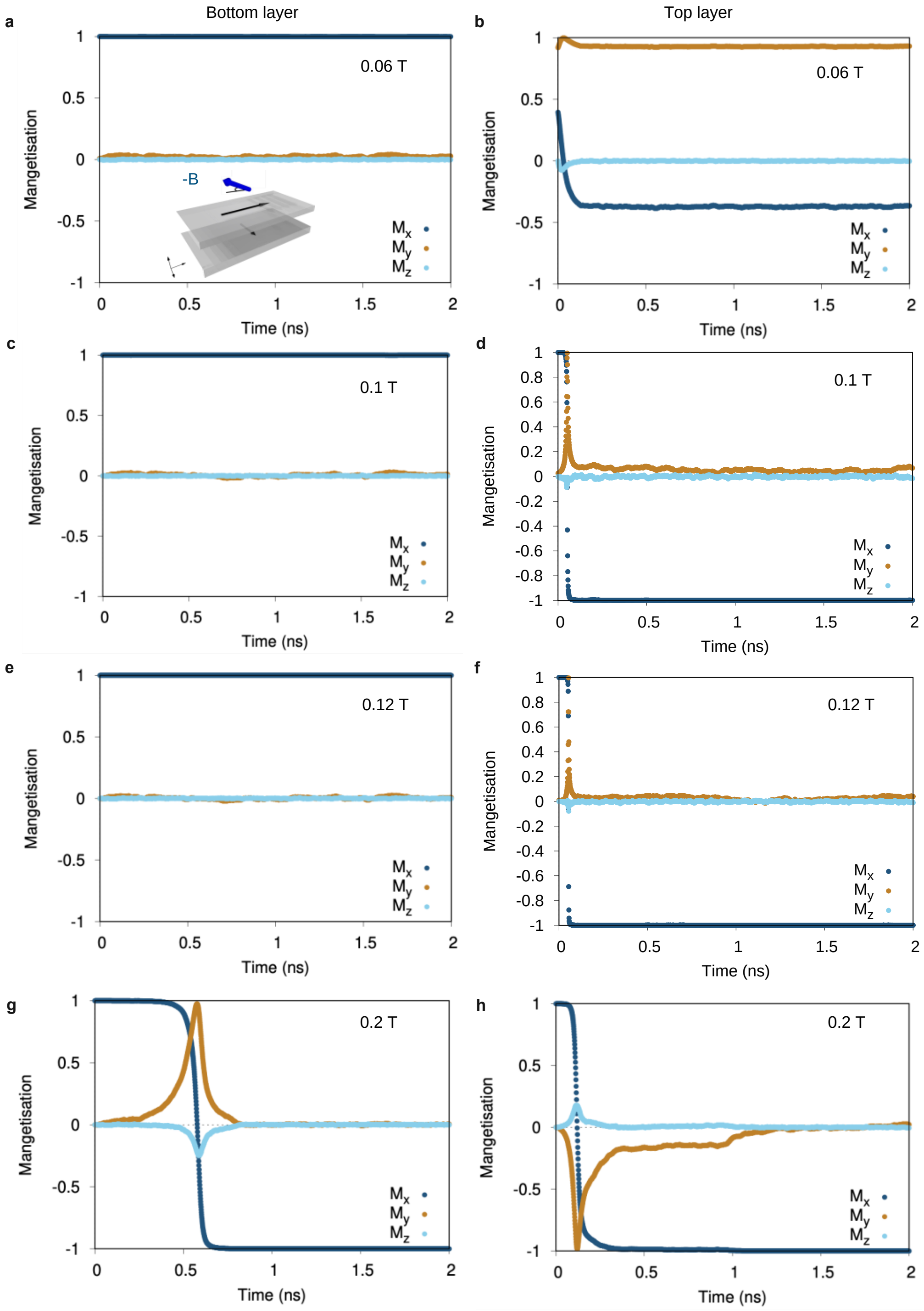

Supplementary Figure 16.- Time relaxation for spin-flip at orthogonally-twisted bilayer CrSBr at reversal field. a-h, Projection of the magnetisation along M_x, M_y and M_z components for the bottom and top layers as function of time. This figure is similar to **Supplementary Figure 15** but with the magnetic field applied along the opposite direction ($-B_x$). This field is anti-parallel and orthogonal to the easy-axis of the bottom and top layers, respectively. A time interval of 2 ns is recorded for the variations of M_x, M_y and M_z under different fields: 0.06 T (**a-b**), 0.1 T (**c-d**), 0.12 T (**e-f**), 0.2 T (**g-h**). The initial spin configurations at 0 ns are those from the simulations with +B showed in **Supplementary Figure 15**. Note that at 0.06 T, the easy-axis of the top layer is tilted between M_x and M_y components since the field is not strong enough to rotate completely the spins. At 0.1 T and 0.12 T a domain wall is initially formed at $+B_x$ situation (**Supplementary Figure 15d,f**) with the average of the spins along the M_x direction. Note that the spins take around 0.2-0.65 ns to change orientation with the applied fields. These finite times induced inhomogeneous magnetic domains and spin textures (**Supplementary Movies S2-S7**) which generate the multi-steps in the magneto-resistance response observed in the devices (**Figure 1**). Since the spins are not totally aligned at intermediate field values at both CrSBr layers, these make the magneto-transport fluctuates abruptly up and down on the resistance. This effect can also be used to approximately quantify how long time the spins take to be fully oriented with the external field.

Section B – Supplementary Movies 1 – 7

Supplementary Movie S1: Multistep magnetization switching with magnetic memory in orthogonally-twisted bilayer CrSBr as shown in **Fig. 3**.

Supplementary Movie S2: Movie of the spin-dynamic simulation at field-cooling of 0.06 mT from above 200 K towards 0 K. The magnetic field is applied following the schematic in the inset of **Fig. 4a** with the field parallel to the easy-axis of the bottom layer. The simulation time comprises 2 ns of the cooling process. An additional 1.5 ns simulation-time is undertaking at 0 K to check further the stability The colour scheme follows that in **Suppl. Fig. S11**.

Supplementary Movie S3: Similar as **Suppl. Movie S2** at an applied field of 0.10 T. The formation of domain-walls occurred at the top layer as it is still rotating to align with the field.

Supplementary Movie S4: Similar as **Suppl. Movie S2** at an applied field of 0.20 T.

Supplementary Movie S5: Similar as **Suppl. Movie S2** with a field of 0.06 mT oriented anti-parallel to the easy-axis of the bottom layer (-B_x).

Supplementary Movie S6: Similar as **Suppl. Movie S5** with a field of $B_x = -0.12$ mT.

Supplementary Movie S7: Similar as **Suppl. Movie S4** with a field of $B_x = -0.2$ mT. Note that in general the formation of the domain walls, spin textures, etc. occurred at the top layer (easy-axis perpendicular to the field) despite the direction of the applied field (±B_x). When the field is reverse (-B_x) however, the magnetic structure of the bottom layer (anti-parallel to the field) becomes more inhomogeneous with more fluctuations of the spins.

Section C – Supplementary Table I

**Intra-monolayer exchange values (meV)**	
J_1	7.2920
J_2	11.0800
J_3	4.4194
J_4	−0.0032
J_5	−0.0537
J_6	−1.1995
J_7	0.4293
**Inter-monolayer exchange values (meV)**	
J_{z1}	−0.0025
J_{z2}	0.0025

Supplementary Table I.- Compendium of the intra and intermonolayer symmetric exchange contributions, J_i, used in atomistic spin dynamics.
